# Wnt5a/β-catenin-mediated epithelial-mesenchymal transition: a key driver of subretinal fibrosis in neovascular age-related macular degeneration

**DOI:** 10.1186/s12974-024-03068-w

**Published:** 2024-03-26

**Authors:** Dandan Liu, Jingxiao Du, Hai Xie, Haibin Tian, Lixia Lu, Chaoyang Zhang, Guo-Tong Xu, Jingfa Zhang

**Affiliations:** 1 Department of Ophthalmology of Tongji Hospital and Laboratory of Clinical and Visual Sciences of Tongji Eye Institute, School of Medicine, Tongji University, Shanghai, China; 2 Department of Ophthalmology, Shanghai General Hospital (Shanghai First People’s Hospital), Shanghai Jiao Tong University School of Medicine, Shanghai, China; 3 Shanghai Key Laboratory of Ocular Fundus Diseases, National Clinical Research Center for Eye Diseases, Shanghai Engineering Center for Visual Science and Photomedicine, Shanghai Engineering Center for Precise Diagnosis and Treatment of Eye Diseases, Shanghai Eye Research Institute, Shanghai, China

**Keywords:** Neovascular age-related macular degeneration, Subretinal fibrosis, Retinal pigment epithelium, Epithelial–mesenchymal transition, Wnt5a/β-catenin

## Abstract

**Background:**

Neovascular age-related macular degeneration (nAMD), accounts for up to 90% of AMD-associated vision loss, ultimately resulting in the formation of fibrotic scar in the macular region. The pathogenesis of subretinal fibrosis in nAMD involves the process of epithelial–mesenchymal transition (EMT) occurring in retinal pigment epithelium (RPE). Here, we aim to investigate the underlying mechanisms involved in the Wnt signaling during the EMT of RPE cells and in the pathological process of subretinal fibrosis secondary to nAMD.

**Methods:**

In vivo, the induction of subretinal fibrosis was performed in male C57BL/6J mice through laser photocoagulation. Either FH535 (a β-catenin inhibitor) or Box5 (a Wnt5a inhibitor) was intravitreally administered on the same day or 14 days following laser induction. The RPE-Bruch's membrane-choriocapillaris complex (RBCC) tissues were collected and subjected to Western blot analysis and immunofluorescence to examine fibrovascular and Wnt-related markers. In vitro, transforming growth factor beta 1 (TGFβ1)-treated ARPE-19 cells were co-incubated with or without FH535, Foxy-5 (a Wnt5a-mimicking peptide), Box5, or Wnt5a shRNA, respectively. The changes in EMT- and Wnt-related signaling molecules, as well as cell functions were assessed using qRT-PCR, nuclear-cytoplasmic fractionation assay, Western blot, immunofluorescence, scratch assay or transwell migration assay. The cell viability of ARPE-19 cells was determined using Cell Counting Kit (CCK)-8.

**Results:**

The in vivo analysis demonstrated Wnt5a/ROR1, but not Wnt3a, was upregulated in the RBCCs of the laser-induced CNV mice compared to the normal control group. Intravitreal injection of FH535 effectively reduced Wnt5a protein expression. Both FH535 and Box5 effectively attenuated subretinal fibrosis and EMT, as well as the activation of β-catenin in laser-induced CNV mice, as evidenced by the significant reduction in areas positive for fibronectin, alpha-smooth muscle actin (α-SMA), collagen I, and active β-catenin labeling. In vitro, Wnt5a/ROR1, active β-catenin, and some other Wnt signaling molecules were upregulated in the TGFβ1-induced EMT cell model using ARPE-19 cells. Co-treatment with FH535, Box5, or Wnt5a shRNA markedly suppressed the activation of Wnt5a, nuclear translocation of active β-catenin, as well as the EMT in TGFβ1-treated ARPE-19 cells. Conversely, treatment with Foxy-5 independently resulted in the activation of abovementioned molecules and subsequent induction of EMT in ARPE-19 cells.

**Conclusions:**

Our study reveals a reciprocal activation between Wnt5a and β-catenin to mediate EMT as a pivotal driver of subretinal fibrosis in nAMD. This positive feedback loop provides valuable insights into potential therapeutic strategies to treat subretinal fibrosis in nAMD patients.

**Supplementary Information:**

The online version contains supplementary material available at 10.1186/s12974-024-03068-w.

## Introduction

Age-related macular degeneration (AMD) is characterized by the progressive degeneration of the neuronal retina, retinal pigment epithelium (RPE) and choriocapillaris complex, leading to severe and permanent central visual impairment among aging population in developed countries [1]. Neovascular AMD (nAMD), accounting for up to 90% of AMD-associated vision loss and blindness [2], is primarily characterized by the presence of choroidal or retinal neovascularization (CNV or RNV) in the macular region, ultimately resulting in the formation of fibrotic scar [2]. The prevalence of fibrosis at baseline, 12, 24, and 60 months in patients with nAMD was reported as follow: 13%, 32%, 36%, and 56%, respectively, according to a recent epidemiological report [3]. Despite the efficacy of anti-vascular endothelial growth factor (anti-VEGF) therapy as the cornerstone in nAMD management, capable of stabilizing or even enhancing the visual function [4], approximately 1/3 of the patients with subretinal fibrosis still experience poor prognosis, and even deterioration of the visual acuity [5]. Therefore, it is imperative and urgent to explore the molecular mechanisms underlying the pathogenesis of subretinal fibrosis to develop effective therapies for subretinal fibrosis secondary to nAMD.

The RPE, a highly specialized monolayer of polarized, pigmented epithelial cells, is located between the outer segment of photoreceptors and the choriocapillaris endothelial cells, playing an essential role in maintaining visual function [6]. The primary functions of RPE encompass light energy absorption focused by the lens onto the retina [7]; transportation of nutrients and ions between photoreceptors and the choriocapillaris [8]; secretion of various factors crucial for maintaining structural integrity in both retina and choriocapillaris [9]; facilitation of phagocytosis in photoreceptor outer segments; and preservation of the blood-retinal barrier [10]. Dysfunction of the RPE, particularly its transition from an epithelial to a mesenchymal phenotype, is implicated in the pathogenesis of subretinal fibrosis, a hallmark of the end-stage complications of nAMD. Recently, several signaling pathways regulating the epithelial–mesenchymal transition (EMT) of RPE cells have been identified, among which the canonical Wnt/β-catenin pathway has been extensively studied and well-characterized [11]. Jung Woo Han et al. demonstrated a crucial role of Wnt/β-catenin signaling in RPE proliferation and EMT using a laser-induced mouse model, while also observing a significant upregulation of the non-canonical Wnt ligand Wnt5a expression in laser-treated RPE compared to control RPE [12]. With accumulating evidence indicating a substantial role for the non-canonical Wnt ligand Wnt5a in the processes of EMT and fibrosis across diverse tissues [13–16], the precise molecular mechanisms underlying Wnt5a-triggered signaling and its interplay with canonical Wnt/β-catenin in EMT, as well as its contribution to the development of subretinal fibrosis in the context of nAMD remain poorly elucidated.

The Wnt signaling pathway plays a crucial role in both development and various diseases. Overall, two primary Wnt signaling pathways can be distinguished: the canonical Wnt/β-catenin pathway, which functions through β-catenin as a transcriptional cofactor, and the non-canonical, β-catenin-independent Wnt pathway, encompassing the Wnt/Ca^2+^ pathway and the planar cell polarity (PCP) pathway [17]. Wnt5a, a highly conserved non-canonical Wnt ligand, functions as a secreted signaling molecule that plays pivotal roles in regulating PCP, convergent extension, and epithelial–mesenchymal interaction during embryonic morphogenesis [18]. Although Wnt5a primarily signals through the non-canonical Wnt signaling pathways by binding to various members of the Frizzled (FZD)- and receptor tyrosine kinase-like orphan receptor (ROR)-family receptors, thereby initiating intracellular signaling cascades, under certain circumstances, it also possessed the capacity to activate the canonical Wnt signaling pathway [19]. This activation led to stabilization of β-catenin and modulation of gene transcription regarding cell proliferation, survival, differentiation, and migration [20]. In the field of cancer biology, the role of Wnt5a exhibits intricate and context-dependent characteristics. Emerging evidence suggests that the dysregulated activation or inhibition of Wnt5a signaling remains an important event in cancer progression, exerting both oncogenic and tumor suppressive effects depending on the availability of key receptors, thereby highlighting the paradoxical role of Wnt5a across different cancers [18]. The involvement of Wnt5a in regulation of cancer cell invasion, metastasis, metabolism and inflammation renders it a subject of intense research in oncology [21]. Furthermore, Wnt5a has been widely recognized as an essential factor in tissue repair and regeneration. For example, in some instances, Wnt5a acting as a particularly attractive growth factor stimulates tissue regeneration (such as colonic crypt [22], osteochondral regeneration [23] and cartilage interface integration [23]) and wound healing [24]. While in other cases, the upregulation of Wnt5a has been implicated in aggravating a variety of tissue fibrosis and scar formation, such as cardiac fibrosis under pressure overload [25], myocardial fibrosis following myocardial infarction [26], atrial fibrosis [25], keloid scarring caused by aberrant genetic activation [13], idiopathic pulmonary fibrosis [27], renal fibrosis [28], and liver fibrosis [29]. However, the specific involvement of Wnt5a in the EMT process of RPE cells and subretinal fibrosis formation in nAMD remains elusive.

Therefore, the objective of this study to elucidate the molecular mechanisms of Wnt5a, a non-canonical Wnt ligand, and its crosstalk with the canonical Wnt signaling pathway involved in EMT of RPE cells, as well as its contribution to subretinal fibrosis progression in nAMD. Additionally, we aim to identify potential therapeutic targets for preventing or halting fibrotic progression in nAMD patients, thereby preserving visual function and improving the quality of life of individuals affected by this devastating disease.

## Materials and methods

### Animals

All mice were housed under specific pathogen-free (SPF) conditions and exposed to a 12-h light/dark cycle with free access to food and water. All procedures were conducted in accordance with the ARVO Statement for the Use of Animals in Ophthalmic and Vision Research and the Guides for the Care and Use of Animals (National Research Council and Tongji University; Permit Number: TJHBLAC-2020–06).

### Scotopic and photopic electroretinography (ERG)

Mice were dark-adapted for a minimum of 12 h before experiments to ensure maximal sensitivity. Prior to ERG recordings, mice were anesthetized by intraperitoneal injection of 0.2 mL 2% pentobarbital sodium. After pupil dilation with compound tropicamide eye drops. ERG recordings were performed in a dark room to ensure minimal ambient light. Gold wire electrodes were carefully placed on the corneas, with the reference electrode positioned under the skin behind the ear. Additionally, a ground electrode was subcutaneously inserted in the upper part of the tail.

For the evaluation of rod photoreceptor function (scotopic ERG), mice were exposed to a dim red light to maintain dark adaptation during electrode placement and recording. Scotopic ERG responses were recorded in response to light stimuli of 0.01 and 3.00 cds/m^2^.

For the assessment of cone function (photopic ERG), mice were exposed to a background light of 25 cds/m^2^ for 5 min, and responses were elicited with flashes of light of 3.00 cds/m^2^. Recorded ERG signals from 6 or 7 eyes were analyzed for amplitude of a-wave and b-wave components.

### Laser-induced CNV mouse model and intravitreal injection

In this study, 7-week-old male C57BL/6J mice were purchased from SLAC Laboratory Animal Co., LTD., Shanghai. The laser-induced CNV model was carried out following the previously established protocol [30]. Briefly, mice were anesthetized by intraperitoneal injection of 0.2 ml 2% pentobarbital sodium and the pupils were dilated with 1% tropicamide (Santen, Osaka, Japan). A 532-nm laser photocoagulation was used to induce CNV in mice (laser power: 120 mW, duration: 100 ms, spot size: 50 μm, Carl Zeiss Meditec; Dublin, Ireland). Four or fifteen laser-induced lesions, resulting in rupture of the RPE and underlying Bruch's membrane, were generated around the optic nerve head in a standardized fashion using a slit lamp delivery system for subsequent immunofluorescence or Western blot analysis on both eyes of each animal.

On the same day or 14 days following laser induction, intravitreal injection of FH535 or Box5 (1 μL) dissolved in dimethyl sulfoxide (DMSO) was administered according to the previous method [31] to reach the target site in the posterior segment of the eye, achieving a final concentration of 0.5/3.0 μmol/L or 90 μmol/L in the vitreous cavity. The contralateral eye received an injection of 1 μL phosphate-buffered saline (PBS) buffer containing 0.03%/0.18% or 5.4% DMSO as a Vehicle control.

### Quantification of the sizes of CNV and subretinal fibrosis

Seven days after laser induction, cardiac perfusion was performed with 4% paraformaldehyde in mice, followed by enucleation of the eyeballs and continued fixation for 30 min. The RPE-Bruch's membrane choriocapillaris complex (RBCC) tissues in each group were isolated and subjected to immunofluorescence staining for fibrosis-related molecules, following the procedure outlined in section “Immunofluorescence”. The immunofluorescence staining results for isolectin B4 (IB4), collagen I, fibronectin, alpha-smooth muscle actin (α-SMA), active β-catenin, and RPE65 were quantified for the measurement of CNV and subretinal fibrosis areas using ImageJ software (National Institutes of Health, Bethesda, MD, USA). Briefly, the images were converted to an 8-bit format. By employing scale calibration and establishing appropriate thresholds, valid fluorescence signals were included for quantitative area measurement, followed by standardized processing.

### Cell culture and treatments

The human RPE cell line (ARPE-19) was obtained from American Type Culture Collection (ATCC, Manassas, VA). ARPE-19 cell line was cultured in Dulbecco's Modified Eagle Medium/Nutrient Mixture F-12 (DMEM/F-12, Hyclone, UT, USA) supplemented with 10% fetal bovine serum (FBS, 40130ES76, Yeasen Biotechnology, Shanghai), 5 U/mL penicillin/streptomycin (60162ES76, Yeasen Biotechnology, Shanghai) and incubated in a humidified atmosphere with 5% CO_2_ at a constant temperature of 37 °C. The ARPE-19 cell line was frequently screened for mycoplasma contamination using an EZ-PCR kit (HaEmek, Israel). Cell culture dishes, palates, and centrifuge tubes were obtained from NEST Biotechnology Co. Ltd. (Wuxi, China).

Recombinant transforming growth factor beta 1 (TGFβ1) was purchased from ThermoFisher (PHG9214, Shanghai, China) and was used at the final concentration of 10 ng/mL. FH535 was obtained from MedChemExpress (MCE, HY-15721, Shanghai) and was used at the final concentration of 0.1, 0.5, 1, and 2 μmol/L respectively. Foxy-5 (Wnt5a agonist) was purchased from MedChemExpress (MCE, HY-P1416A, Shanghai) and was used at the final concentration of 50, 100, and 200 μmol/L, respectively. Box5 (Wnt5a antagonist) was purchased from MedChemExpress (MCE, HY-123071, Shanghai) and was used at the final concentration of 10, 45, and 90 μmol/L. The peptides were purified by reverse-phase high performance liquid chromatography and the purity of Box5 was 91%, and the purity of FH535 and Foxy-5 was more than 99%. The human ARPE-19 cell line was subjected to serum-free starvation for 12 h, followed by treatment with TGFβ1 or combination with above small molecules in fresh serum-free medium (SFM) for an additional 48 h to investigate the impact of Wnt signaling on EMT.

### Plasmid-mediated Wnt5a shRNA transfection in ARPE-19 cells

The short hairpin RNA (shRNA) was employed to knockdown Wnt5a to explore the effect of Wnt5a on the EMT of ARPE-19 cells in vitro. Briefly, ARPE-19 cells were seeded in appropriate culture dishes or plates and allowed to reach 50–60% confluency. The plasmids containing Wnt5a shRNA1 or shRNA2 was introduced into the cells using a transfection reagent Lipofectamine 3000 according to the manufacturer's instructions (Beijing Tsingke Biotech Co., Ltd.). Optimal transfection conditions, including plasmid-to-reagent ratio (1:1) and incubation time (36 h), were determined through optimization experiments. Following transfection, the effectiveness of Wnt5a knockdown was assessed using quantitative real-time polymerase chain reaction (qRT-PCR) and Western blot analysis. The sequences targeting Wnt5a knockdown were 5′- CCGGGCTGGAAGTGCAATGTCTTCCCTCGAGGGAAGACATTGCACTTCCAGCTTTTTT-3′ (Wnt5a shRNA1), and 5′-CCGGGGTCGCTAGGTATGAATAACCCTCGAGGGTTATTCATACCTAGCGACCTTTTTT-3′ (Wnt5a shRNA2, Tsingke, Beijing, China). The ARPE-19 cells exhibiting successful knockdown of Wnt5a gene will be selected for subsequent experiments.

### Cell viability assay

Cell viability was measured by CCK-8 kit (40203ES76, Yeasen, Shanghai, China) according to the manufacturer's protocol. A total of 5 × 10^3^/well cells were seeded uniformly and cultured in five replicate wells in a 96-well microplate (Corning, USA) with medium containing 10% FBS, and then were incubated at 37 °C, 5% CO_2_ incubator for 2 days. Afterwards, the culture medium was replaced with SFM for 12 h of starvation treatment. Then, the cells were exposed to varying concentrations of FH535 or Box5, dissolved in DMSO, or Foxy-5 dissolved in PBS, all within SFM. The Vehicle Control groups for FH535 and Box5 contained DMSO concentrations of 0.2% and 2%, respectively. After treatment for 48 h, the medium was discarded. The CCK-8 reagent (10 μL) was added to 90 μL SFM to generate a working solution (total 100 μL) and then added to each well and incubated for 1–2 h. A microplate reader (Bio‐Rad, Hercules, CA, USA) was employed to test the absorbance of each experimental well at 450 nm, and to detect changes of cell viability among each group.

### Scratch assay

ARPE-19 cells were uniformed seeded in 6-well plates (1 × 10^6^ cells per well) and cultured for 2 days till the desired confluency. ARPE-19 cells were then deprived of serum for 12 h. Migration was evaluated by straight scratching a confluent layer of ARPE-19 cells using a P200 pipette tip. After gently washing with serum-free media to remove cell debris, 2 mL of SFM with or without TGFβ1 [10 ng/mL], FH535 [0.5 μmol/L], Box5 [90 μmol/L], or Foxy-5 [200 μmol/L] was added, followed by incubation at 37 °C. Images were obtained at 0, 24 and 48 h under a fluorescence microscope (Leica, DMI3000, Germany), after which the reduction in the wound area was determined using Image‐Pro Plus software (Media Cybernetics, Rockville, MD, USA).

### Transwell migration assay

1 × 10^6^ cells/well of ARPE-19 cells were uniformly seeded in 6-well plates. After 48 h of TGFβ1 treatment with or without FH535 co-incubation. ARPE-19 cells were trypsinized and then resuspended in SFM. 1 × 10^4^ cells/well of ARPE-19 cells were seeded into the upper compartment of transwell cell culture chambers (8-µm pores; Falcon; Corning Life Sciences, Corning, NY, USA). 500 µL of complete DMEM/F12 medium was added to the lower inserts of the transwell. After incubation for 48 h at 37 °C, cells on the upper compartment of the transwell were removed by using a swab and cells across pores were fixed with cold methanol for 10 min and stained with crystal violet stain solution (0.5%, Beyotime Biotechnology, Haimen, China)) for 1 h. Images of migrated RPE cells were taken under the Leica microscope (DMI3000, Germany) and were quantified with the help of Image J software. The number of migrated cells in more than 12 random fields (magnification × 20) was counted.

### qRT-qPCR

Total RNA was extracted from ARPE-19 cells using TRIzol Reagent (19201ES60, Yeasen; Shanghai, China) according to the manufacturer’s instructions. Complementary DNA was synthesized using a reverse transcription SuperMix (11120ES60, Yeasen; Shanghai, China). The SYBR Green master mix (11201ES03, Yeasen; Shanghai, China) was employed to conduct RT-qPCR. The conditions were set as follows: 95 °C for 5 min, 40 cycles of 95 °C for 10 s, 60 °C for 20 s and extension at 72 °C for 20 s. Relative expression of mRNA was calculated using the 2^−ΔΔCt^ method [32] and normalized to glyceraldehyde-3-phosphate dehydrogenase (GAPDH). Primer sequences are listed in Table 1.

**Table 1:** The information of human primers

Gene	Forward (5′-3′)	Reverse (5′-3′)
α-SMA	CAGAAGGAGATCACGGCCCTAG	CGGCTTCATCGTATTCCTGTTTG
collagen I	AATGTGGTTCGTGACCGTGA	AGCCTTGGTTGGGGTCAATC
Dvl2	TGGGGCTTCAGACCAGGATA	GCCCCCACCATAGGTGTAAG
Dvl3	TGCTGATAACCCATCGGAGC	TGGGCAGACACCAAAGAGTC
fibronectin	AAGACCATACCCGCCGAATG	GGCATTTGGATTGAGTCCCG
FZD1	GAAAGTGCAGTGTTCCGCTG	TCACACTTGAGCGTGTCTGG
FZD2	CTATCCGCTGGTGAAGGTGC	CTCACAGATAGAGCGGCACGG
FZD3	TGATGGCTCTCATAGTTGGCA	ACCTGTCGGCTCTCATTCAC
FZD4	GTCTCAGTCTGGGGTTGCTC	ACGTTGTAGCCGAGGTTCTG
GAPDH	CAAATTCCATGGCACCGTCA	GACTCCACGACGTACTCAGC
ITGA5	GGTCGGGGGCTTCAACTTA	AGCACACTGACCCCGTCTG
MMP2	TGATGGCATCGCTCAGATCC	GGCCTCGTATACCGCATCAA
NAKED1	AGGGGGAATAGGTGAGACCC	GGAATCCCATTGGCTGGTCA
NAKED2	CTTTCCGGGAGGACCAGTGT	GCACTGGAGTGCGTCAATGT
Snail1	TGCAGGACTCTAATCCAGAGTTT	GGACAGAGTCCCAGATGAGC
Transgelin	AAGCGCAGGAGCATAAGAGG	ACTGATGATCTGCCGAGGTC
Wnt1	CAAGATCGTCAACCGAGGCT	TCACACGTGCAGGATTCGAT
Wnt3a	ACAAAGCTACCAGGGAGTCG	TCCCACCAAACTCGATGTCC
Wnt5a	CGGTGTACAACCTGGCTGAT	GCGCTGTCGTACTTCTCCTT

### Western blot

Total cell lysates and samples of mouse RBCC tissues were lysed with radio immunoprecipitation assay lysis buffer (RIPA, 20101ES60, Yeason, Shanghai, China) on ice and sonicated for 10 s immediately. The cytoplasmic and nuclear extracts of ARPE-19 cells were prepared using the ProteinExt^®^ Mammalian Nuclear and Cytoplasmic Protein Extraction Kit (DE20101, transgen, Beijing, China), according to the manufacturer’s instructions. Lamin B1 and GAPDH were employed as nuclear and cytoplasmic loading controls, respectively.

The samples were centrifuged at 4 °C at 12,000 rpm for 30 min and the supernatant was collected for bicinchoninic acid assay (20201ES76, Yeason, Shanghai, China) and Western blot as previously described [31]. Briefly, protein was denatured and resolved in 7.5–12.5% SDS-PAGE gels and transferred onto nitrocellulose membranes. The blots were blocked for 30 min at room temperature with 5% bovine serum albumin (BSA) in tris-buffered saline with Tween-20 (TBST) and then probed with fibronectin, α-SMA, zonula occludens-1 (ZO-1), Wnt3a, Wnt5a, ROR1, dishevelled 2 (Dvl2), Naked1, non-phospho (active) β-catenin (Ser33/37/Thr41), lamin B1 and GAPDH overnight at 4 °C separately. After thorough washing with TBST solution four times for 6 min each time, the corresponding secondary antibodies (goat anti-mouse/rabbit) conjugated to HRP were employed to incubate the blots for 1 h at room temperature. The bands were washed by TBST for 4 times and then visualized by chemiluminescence. Quantification was performed by measuring the relative intensity of interested signals which normalized by GAPDH or lamin B1 with the aid of Quantity One software (Bio-Rad). The primary antibodies are listed in Table 2.

**Table 2:** Antibody information

Target	Catalogue no	Application (Conc.)	Vendor
α-SMA	Ab7817	WB (1:2,000); IF (1: 100)	Abcam
collagen I	Ab34170	WB (1:1,000); IF (1: 100)	Abcam
Dvl2	3224	WB (1:1,000)	CST
fibronectin	15,613-1-AP	WB (1:500); IF (1: 100)	Proteintech
GAPDH	10,494-1-AP	WB (1: 5,000)	Proteintech
IB4	I21411/I32450	IF (1:2000)	Invitrogen
lamin B1	AF5161	WB (1:500)	Affinity
Naked1	2262	WB (1:1,000)	CST
Non-phospho (Active) β-Catenin (Ser33/37/Thr41)	8814	WB (1:1,000); IF (1:800)	CST
ROR1	4102	WB (1:1,000)	CST
RPE65	Ab13826	IF (1: 100)	Abcam
vimentin	10,366-1-AP	IF (1: 100)	Proteintech
Wnt3a	2721	WB (1:1,000)	CST
Wnt5a	2392	WB (1:1,000)	CST
ZO-1	61–7300	WB (1:1,000); IF (1: 100)	Invitrogen

WB, Western blot; IF, immunofluorescence; CST, Cell Signaling Technology

### Immunofluorescence

The ARPE-19 cells and mouse RBCC were fixed with 4% paraformaldehyde (PFA) (Servicebio, Wuhan, China) for 20 min, permeabilized with 0.05% Triton X-100 for 20 min, and then blocked with 1% BSA for 1 h. The slides were incubated overnight with primary antibodies against collagen I, fibronectin, α-SMA, vimentin, RPE65, ZO-1, or active β-catenin overnight, followed by IB4 probe, Alexa Fluor 488/555 goat anti-mouse or Alexa Fluor 555/488 goat anti-rabbit conjugated secondary antibodies, respectively, at room temperature in the dark for 1 h. The nucleus was visualized with 4′,6-diamidino-2-phenylindole (DAPI). The slides were mounted with coverslips. Images were obtained with a confocal microscope from Carl Zeiss (LSM 710; Königsallee, Germany).

### Statistical analysis

Data generated in this study are presented as means ± SEM from at least three independent experiments. Statistical analysis was performed using GraphPad Prism 8.0 software (San Diego, CA, USA). For the comparison between the two experimental groups and the intergroup comparison, we applied two-tailed unpaired Student's *t*-test or one-way analysis of variance (ANOVA) as appropriate. Differences were considered statistically significant when *P*-values are less than 0.05.

## Results

### FH535 mitigated the dimensions of CNV and subretinal fibrosis, along with the EMT of RPE cells in the laser-induced CNV mouse model

Wnt signaling plays pivotal roles in promoting myofibroblast activation, EMT, and tissue fibrotic processes in age-related diseases [33]. To further validate the potential involvement of Wnt signaling pathways in promoting subretinal fibrosis, we initially established a laser-induced CNV mouse model, which serves as an appropriate experimental platform for studying subretinal fibrosis since there are currently no successful animal models of subretinal fibrosis secondary to nAMD [34]. Subsequently, we investigated the role of FH535, a novel β-catenin/T-cell factor (TCF) antagonist [35], in this mouse model.

**Fig. 1:**
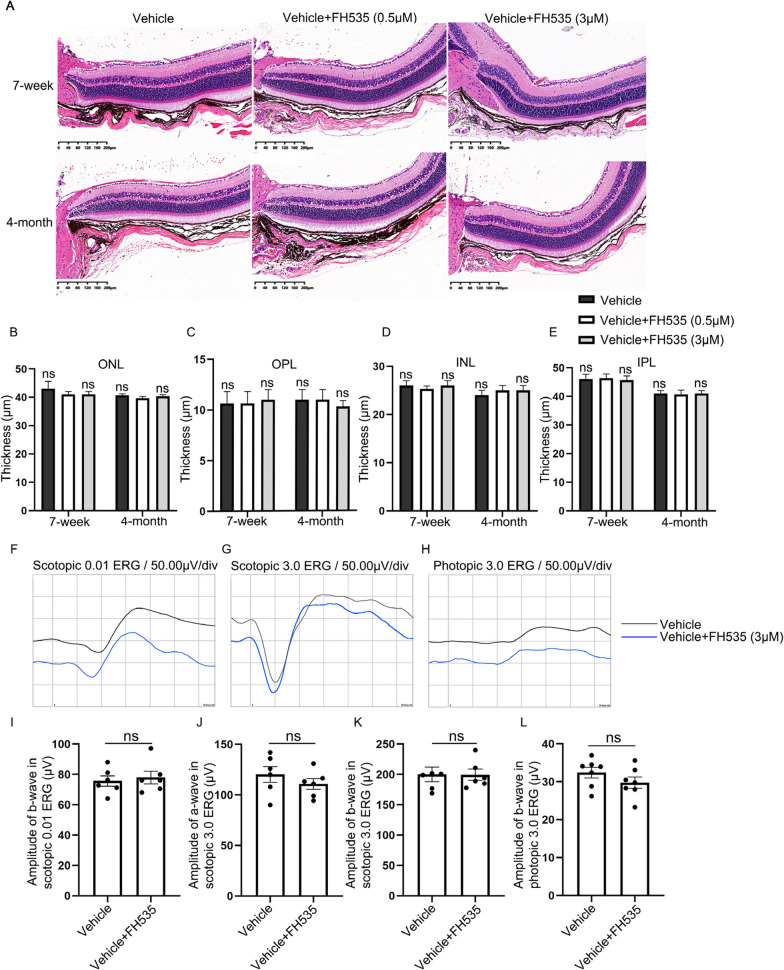
Safety assessment of intravitreal administration of FH535 in C57 mice. **A** Hematoxylin eosin (HE) staining of the retinas in 7-week and 4-month mice was shown after intravitreal injection of different concentrations of FH535 (0.5 or 3.0 μmol/L) for 7 days. **B**–**E** Quantitative analysis of ONL, OPL, INL, and IPL thickness in retinas among abovementioned groups. **F**–**H** Representative results of scotopic ERG at 0.01 or 3.0 log cds/m^2^ and photopic ERG at 3.0 log cds/m^2^ in 4-month-old male C57BL/6J mice 7 days after intravitreal injection of FH535 (3.0 μmol/L), compared to the Vehicle group injected with PBS. **I** The mean scotopic ERG b-wave amplitudes elicited by 0.01 log cds/m^2^ white-light stimuli. **J** The mean scotopic ERG a-wave amplitudes elicited by 3.0 log cds/m^2^ white-light stimuli. **K** The mean scotopic ERG b-wave amplitudes elicited by 3.0 log cds/m^2^ white-light stimuli.   The mean photopic ERG b-wave amplitudes elicited by 3.0 log cds/m^2^ white-light stimuli. *Scale bars*, 200 μm. ERG, Electroretinography; INL, inner nuclear layer; IPL, inner plexiform layer; OPL, outer plexiform layer; ONL, outer nuclear layer. Data are expressed as mean ± SEM. ns, no significant

The safety of intravitreal injection of FH535 was validated by the hematoxylin and eosin (H&E) staining, as well as scotopic and photopic ERG. The H&E staining results depicted in Fig. 1A–E indicated that, after intravitreal injection of 0.5 μmol/L or 3 μmol/L FH535 for 7 days in male C57BL/6J mice aged 7 weeks or 4 months, there were no significant differences observed in the thickness of multiple retinal nuclear layers, including the outer nuclear layer (ONL), outer plexiform layer (OPL), inner nuclear layer (INL), and inner plexiform layer (IPL), compared to the Vehicle group injected with PBS (0.03% or 0.18% DMSO). Furthermore, the effects of intravitreal injection of FH535 (3 μmol/L) on mice retinal function were investigated by using scotopic and photopic ERG. The results indicated that intravitreal injection of FH535 (3 μmol/L) had no significant impact on retinal function in mice aged 4 months, compared to the Vehicle group injected with PBS (0.18% DMSO), seven days post-injection (Fig. 1F–L). In this study, FH535 at a relatively low concentration of 0.5 μmol/L was selected for further study.

**Fig. 2:**
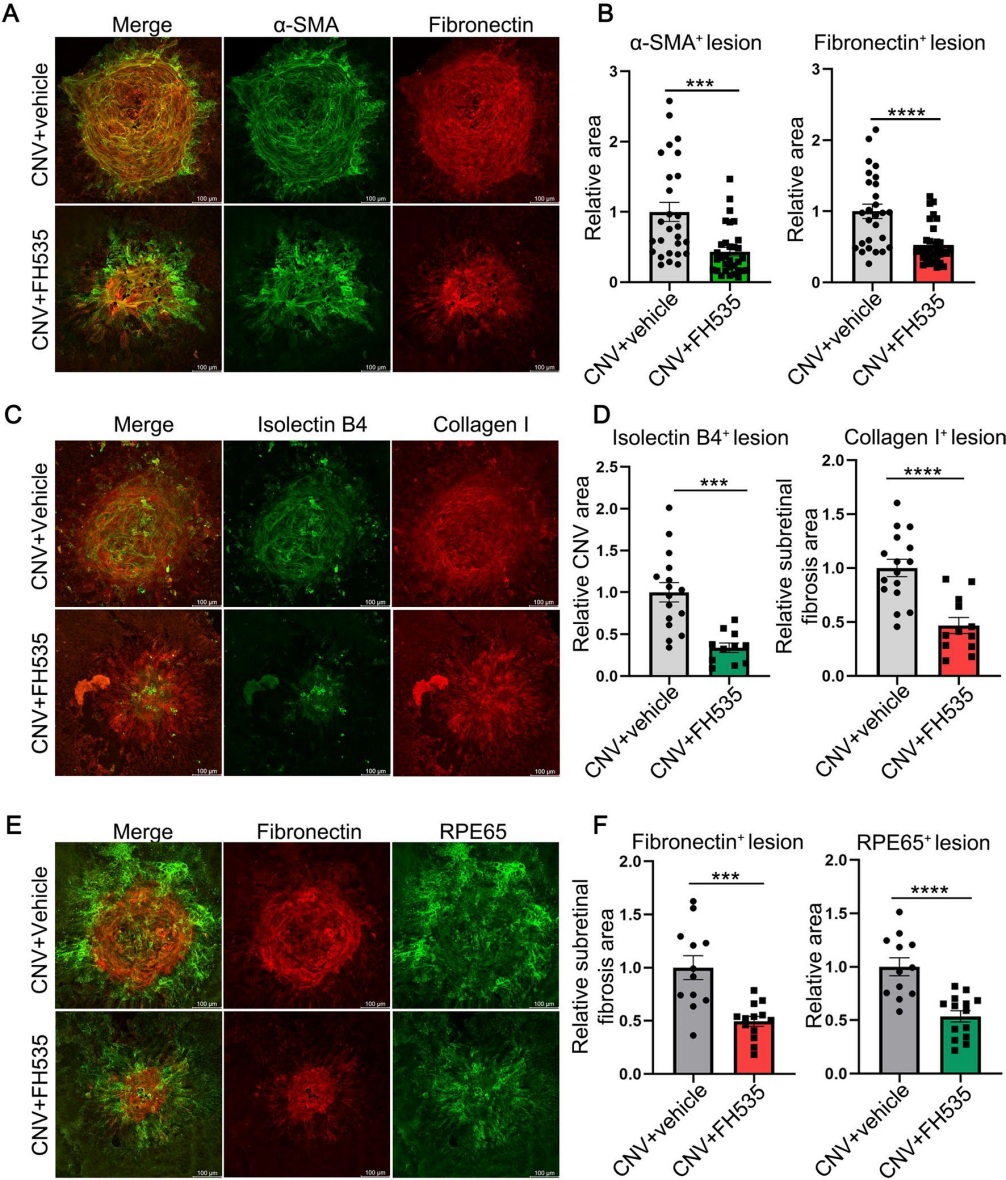
The effects of FH535 on subretinal fibrosis, EMT and CNV in laser-induced CNV mice. **A**, **C** Immunofluorescence of the whole RBCC flat-mounts showing intravitreal injection of FH535 (0.5 μmol/L) inhibited the degree of subretinal fibrosis and CNV in mice at day 7 post laser induction. The fibrosis and neovascularization were marked by α-SMA, fibronectin, collagen I and IB4. **B**, **D** Quantitative analysis of the effects of FH535 on the subretinal fibrotic area and neovascularization area in laser-induced CNV mice. **E** Immunofluorescence staining of the whole RBCC flat-mounts showing the effects of FH535 on the co-staining of fibrotic indicator fibronectin and RPE marker RPE65 in CNV model mice. **F** Quantitative analysis of the effects of FH535 on the subretinal fibrotic area and RPE65-positive immunofluorescence area in CNV model mice. *Scale bar*s, 100 µm. α-SMA, alpha-smooth muscle actin; CNV, choroidal neovascularization. Data are expressed as mean ± SEM. ***p < 0.001, ****p < 0.0001 compared with the CNV group

To assess the potential inhibitory effects of FH535 on EMT, subretinal fibrosis, and CNV in the laser-induced mouse model at Day 7, we performed immunostaining for fibrotic marker (fibronectin), extracellular matrix (ECM) protein (collagen type I [collagen I]), mesenchymal cell marker (α-SMA), and endothelial cell marker (IB4). As shown in Fig. 2A–D, FH535 treatment markedly decreased the immunostaining areas of fibronectin by 52.80% (*p* = 0.00004, n = 27 or 32), α-SMA by 43.46% (*p* = 0.00017, n = 27 or 32), collagen I by 46.82% (*p* = 0.00008, n = 16 or 12) and IB4 by 34.74% (*p* = 0.00016, n = 16 or 11) in RPE-choroid complexes of CNV group compared to the control group receiving intravitreal injections of PBS (0.03% DMSO), indicating that blockage of Wnt/β-catenin by FH535 could effectively decrease both subretinal fibrosis and neovascularization in CNV mice.

To elucidate the role of EMT implicated in the formation of subretinal fibrosis secondary to CNV, as well as the impact of FH535 on this process, immunostaining for RPE65 (a specific marker for RPE cells) and fibronectin was performed in laser-induced CNV mice. As shown in Fig. 2E, in the CNV group, RPE cells exhibited robust expression of RPE65 protein, particularly surrounding and displaying a propensity toward the fibronectin-positive fibrotic lesions, indicative of the progression of EMT and myofibroblast transformation from the RPE cells; however, this phenotype was largely attenuated after FH535 treatment. Compared with the laser-induced CNV mice, the area of transformed RPE was evidently reduced by 52.57% (*p* = 0.00007, n = 12 or 16, Fig. 2E and F) following intravitreal injection of FH535. The presence of transformed RPE cells surrounding pathological CNV was also observed in Sprague–Dawley rats through retinal cryosection after Bruch membrane’s rupture due to subretinal injection to induce pathological neovascularization (Additional file 1: Fig. S1). The co-localization of RPE65 and fibronectin in subretinal fibrosis lesions suggested the EMT of RPE within CNV lesions might play a vital role in the initiation and development of subretinal fibrosis.

### Wnt5a and active β-catenin are key molecules promoting RPE cell EMT and the subsequent subretinal fibrosis process in nAMD

To elucidate the underlying mechanisms by which FH535 suppressed subretinal fibrosis and EMT through modulation of Wnt signaling, we investigated the alterations in Wnt-related molecules, including Wnt5a, ROR1, Wnt3a, and β-catenin, in RBCC tissues obtained from CNV mice treated with or without FH535.

**Fig. 3:**
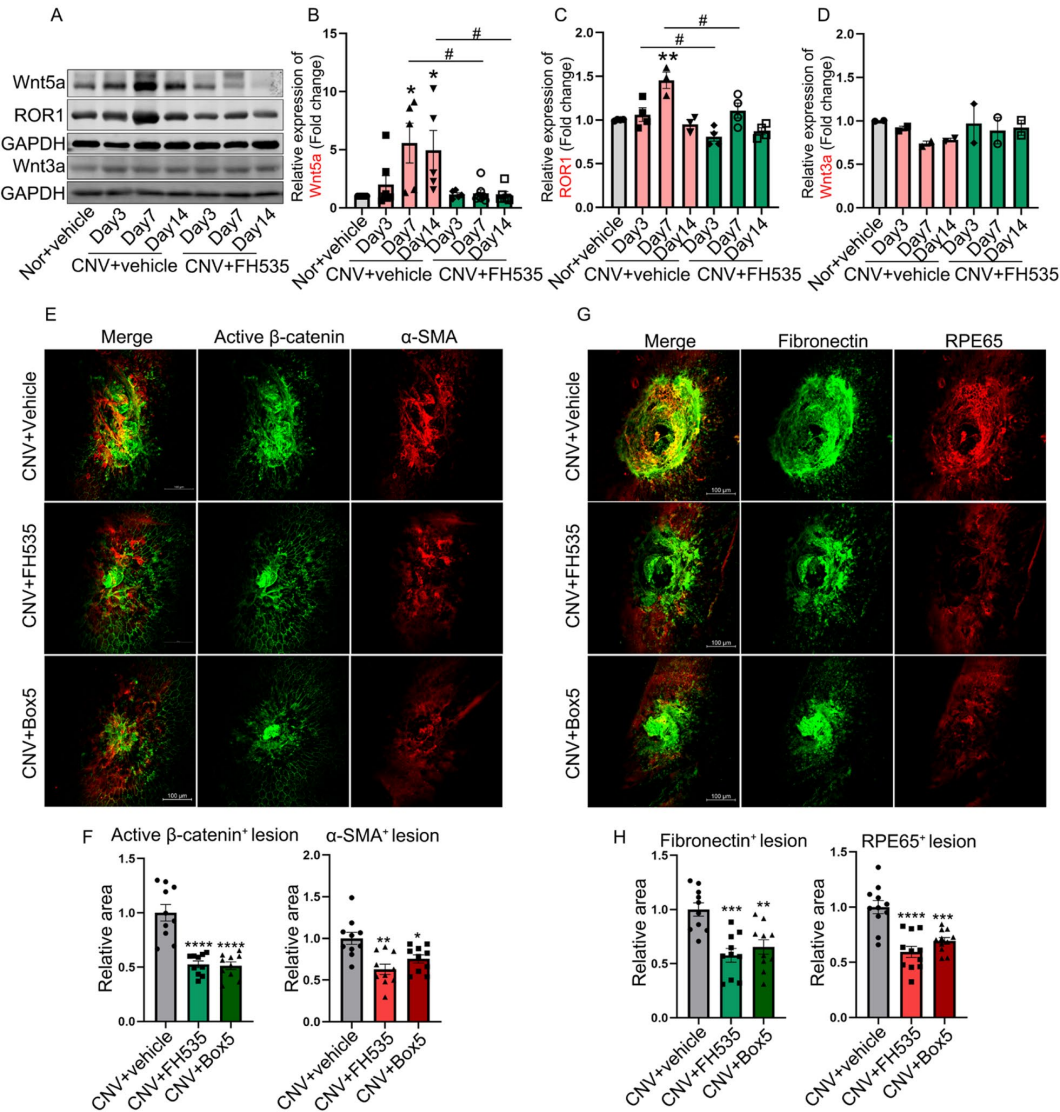
The impact of intravitreal administration of FH535 or Box5 on Wnt-signaling, EMT and subretinal fibrosis in laser-induced CNV mice. **A**–**D** The changes of Wnt-related molecules (Wnt5a, ROR1, and Wnt3a) examined with Western blot in RBCCs from day 3 to day 14 after laser induction in mice with or without FH535 treatment (0.5 μmol/L). **E** and **F** The effects of intravitreal injections of FH535 or Box5 (90 μmol/L) on β-catenin and α-SMA expression within the CNV lesions in mice at day 21 after laser induction, along with the corresponding quantitative analysis. **G** and **H** The immunofluorescence of fibronectin and RPE65 in RBCC flat-mounts of mice among above different groups. *Scale bar*s, 100 µm. α-SMA, alpha-smooth muscle actin; CNV, choroidal neovascularization; Nor, Normal; ROR1, receptor tyrosine kinase-like orphan receptor 1; RBCC, RPE-Bruch’s membrane-choriocapillaris complex. Data are expressed as mean ± SEM. *p < 0.05, **p < 0.01, ***p < 0.001, ****p < 0.0001 compared with the Nor or CNV group; ^#^*p* < 0.05 compared with CNV group (**B** and **C**)

The Western blot results depicted in Fig. 3 indicated that, compared with normal tissues, the protein levels of Wnt5a and ROR1 were significantly up-regulated, while Wnt3a remained relatively unchanged (Fig. 3A–D), suggesting the involvement of non-canonical Wnt ligand Wnt5a in the pathogenesis of CNV-associated subretinal fibrosis. Furthermore, the results illustrated that intravitreal administration of FH535 on the same day following laser photocoagulation significantly suppressed the expression of Wnt5a and ROR1, while no significant effect was observed on Wnt3a level, when compared to those in the PBS injection group. The presented data strongly implied that the inhibition of Wnt/β-catenin by FH535 notably suppressed the exaggerated expression of Wnt5a and ROR1 in CNV mice.

To further investigate the involvement of Wnt5a and β-catenin in the process of subretinal fibrosis induced by laser in mice, we administered intravitreal injections of the Wnt5a antagonist Box5 (90 μmol/L) or FH535 (0.5 μmol/L) at a late time point (Day 14). This specific time point was chosen to avoid the initial neovascularization stage in this mouse model, thereby the influence of neovascularization on subretinal fibrosis can be excluded, enabling a precise elucidation of the anti-fibrotic effects exerted by Box5 or FH535. Based on the ERG results depicted in Additional file 1: Fig. S4, our findings indicated that intraocular administration of Box5 (90 μmol/L) exhibited a favorable safety profile.

Seven days following intravitreal injections (Day 21) in CNV mice, RBCC samples were subjected to immunofluorescence staining analysis for active β-catenin, α-SMA, fibronectin and RPE65. As shown in Fig. 3, compared to the Vehicle treatment group, FH535 (0.5 μmol/L) treatment markedly decreased the immunostaining areas of active β-catenin by 47.39% (*p* = 0.00002, n = 10, Fig. 3E and F), α-SMA by 37.02% (*p* = 0.0010, n = 10, Fig. 3E and F), fibronectin by 42.27% (*p* = 0.00016, n = 10, Fig. 3G and H), RPE65 by 40.60% (*p* = 0.00004, n = 10, Fig. 3G and H); while Box5 (90 μmol/L) treatment significantly reduced the immunostaining areas of active β-catenin by 47.39% (*p* = 0.00002, n = 10, Fig. 3E and F), α-SMA by 24.62% (*p* = 0.0114, n = 10, Fig. 3E and F), fibronectin by 34.60 (*p* = 0.0013, n = 10, Fig. 3G and H), RPE65 by 30.60% (*p* = 0.00017, n = 10, Fig. 3G and H), in RPE-choroid complexes of CNV mice. The in vivo data presented herein suggested that Wnt5a and active β-catenin play pivotal roles in driving EMT of RPE cells and subsequent subretinal fibrosis. Furthermore, a reciprocal dependence or activation between Wnt5a and β-catenin was observed during this process.

### The Wnt signaling molecules, particularly Wnt5a and active β-catenin, exhibit an upregulation in TGFβ1-treated ARPE-19 cells

**Fig. 4:**
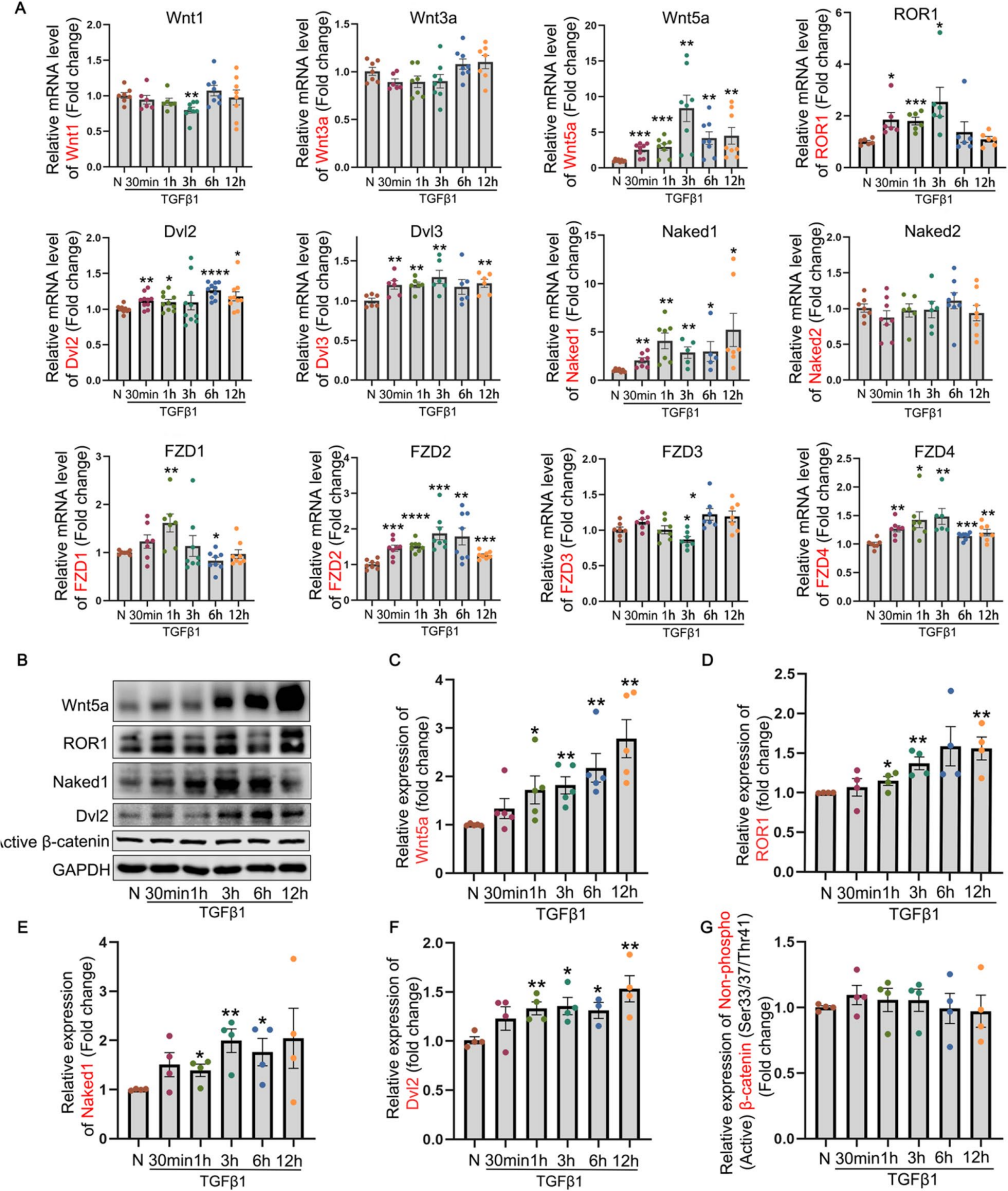
The influence of TGFβ1 on the Wnt-signaling molecules in ARPE-19 cells. The expression of Wnt signaling molecules was assessed by **A** RT-qPCR and **B**–**G** Western blot in ARPE-19 cells treated with TGFβ1 (10 ng/mL) for different time points within a time duration of 12 h. Dvl, dishevelled; FZD, frizzled receptor; h, hour (s); min, minutes; N, normal; ROR1, receptor tyrosine kinase-like orphan receptor 1; TGFβ1, transforming growth factor beta 1. All results performed above are presented as mean ± SEM from three independent experiments. *p < 0.05, **p < 0.01, ***p < 0.001, ****p < 0.0001 compared with untreated ARPE-19 cells

Next, we verified the aforementioned findings in a cellular model of EMT induced by TGFβ1 in ARPE-19 cells. First, the key signaling molecules associated with the canonical and non-canonical Wnt signaling pathways were investigated by qRT-PCR and Western blot analyses to determine whether these molecules are activated in TGFβ1-treated ARPE-19 cells. The results presented in Fig. 4 demonstrated a significant upregulation of Wnt5a and ROR1, along with other key molecules involved in Wnt signaling (Dvl2, Naked1 and FZD1-4), while the remaining components of the Wnt signaling (Wnt1, Wnt3a, active β-catenin, and Naked2) remained unchanged in TGFβ1-treated ARPE-19 cells within a 12-h timeframe compared to the control group, as detected by qRT-PCR (Fig. 4A) and Western blot (Fig. 4B–G). However, the findings presented in Fig. 6A and D demonstrated a significant increase in the level of active β-catenin following a prolonged 48-h treatment with TGFβ1 in ARPE-19 cells.

### Inactivation of Wnt/β-catenin signaling by FH535 attenuated the EMT and migratory behaviors of TGFβ1-treated ARPE-19 cells

Then, we assessed the impact of FH535 on the activation of EMT in TGFβ1-induced ARPE-19 cells. Firstly, we conducted a CCK-8 cytotoxicity assay to determine the non-toxic dose range of FH535 (0–5 μmol/L) in ARPE-19 cells (Additional file 1: Fig. S2A). The subsequent results demonstrated the mRNA and protein expression levels of EMT-related markers in TGFβ1-treated ARPE-19 cells were significantly increased, when compared to untreated control cells, which were largely attenuated by FH535 in a dose-dependent manner (Additional file 1: Fig. S3A-H). Conversely, the tight junction protein ZO-1 exhibited a significant reduction in the TGFβ1-treated group compared to the Vehicle-treated group, while which was definitely preserved following FH535 treatment, suggesting that incubation of FH535 confers protection to cellular tight junction (Additional file 1: Fig. S3I). After considering the aforementioned results, we ultimately opted for a concentration of 0.5 μmol/L for subsequent experiments.

**Fig. 5:**
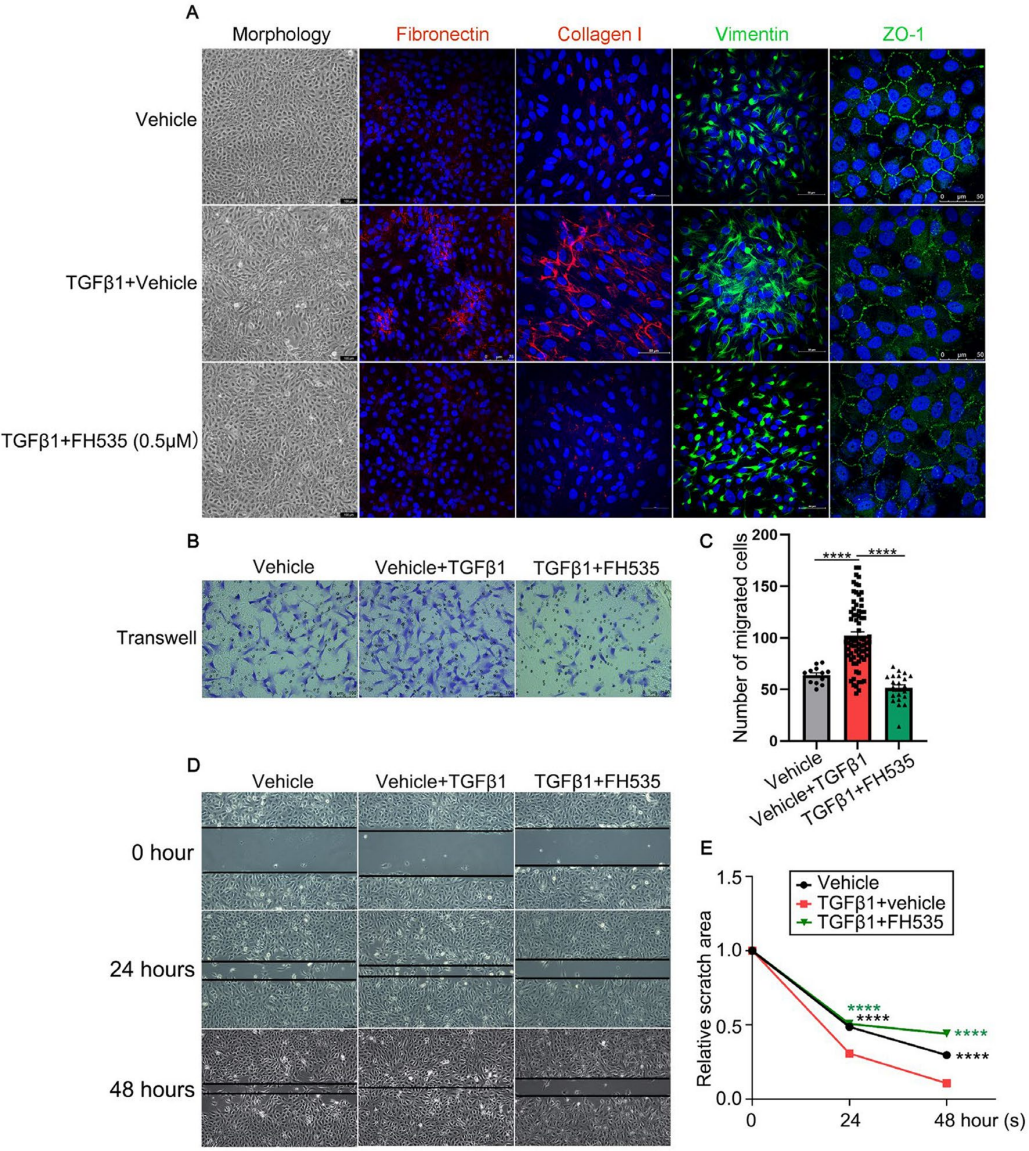
The impact of FH535 co-incubation on the EMT and migratory capacity of ARPE-19 cells treated with TGFβ1. **A** Representative bright field images depicting cellular morphology, as well as immunostainings for fibronectin, collagen I, vimentin and ZO-1 proteins in ARPE-19 cells among Vehicle (0.005% DMSO), TGFβ1 (10 ng/mL) + Vehicle (0.005% DMSO), and TGFβ1 + FH535 (0.5 μmol/L) groups after treatment for 48 h. DAPI (blue). *Scale bars* = 100, 75 or 50 μm. **B** Representative images and **C** quantitative analysis of the number of migrating ARPE-19 cells subjected to the transwell migration assay after treatment of TGFβ1 with or without FH535 for 48 h. *Scale bar* = 100 µm. **D** Representative images depicting the scratch wound healing assay of ARPE-19 cells were captured at 0 h, 24 h, and 48 h after incubation of TGFβ1 in the presence or absence of FH535. The black lines showed the edge of the wound. **E** Statistical graph of scratch wound healing assay of ARPE-19 cells among above different groups. TGFβ1, transforming growth factor beta 1; ZO-1, zonula occludens-1. All results performed above are presented as mean ± SE from three independent experiments. ****p < 0.0001 compared with the TGFβ1-treated group

In parallel with the data of qRT-PCR and Western blot, immunostaining of both collagen I and fibronectin, as well as vimentin validated that FH535 treatment was able to reduce the fibrosis-related markers caused by TGFβ1 in ARPE-19 cells (Fig. 5A). Immunofluorescence analysis also revealed that the downregulated expression and disrupted localization of ZO-1 in TGFβ1-induced ARPE-19 cells were effectively reversed following treatment with FH535 (Fig. 5A). Additionally, The EMT phenotype of ARPE-19 cells following TGFβ1 treatment was further authenticated based on the characteristic morphological changes, exhibiting a stretched and elongated appearance, which was subsequently reversed upon co-incubation with FH535 (Fig. 5A).

Since the significant correlation between the EMT phenotype and the enhanced migratory capacity of ARPE-19 cells, the transwell migration assay and scratch wound-healing assay were performed to evaluate the impact of FH535 on the invasion and migration ability of TGFβ1-treated ARPE-19 cells. The transwell migration assay revealed a significant increase of 160.53% (n = 12 and 73, *p* < 0.0001) in TGFβ1-induced cell migration compared to the normal group, whereas co-treatment with FH535 significantly inhibited cell migration and invasion in TGFβ1-treated ARPE-19 cells (Fig. 5B and C, n = 21, *p* < 0.0001). Wound-healing assay demonstrated that TGFβ1 effectively enhanced the cell migration and wound closure in ARPE-19 cells, resulting in a significant reduction to 30.72% at 24 h and 10.62% at 48 h in the wound area compared to the control group (n > 20, *p* < 0.00001, Fig. 5D and E). However, the area of wound closure in TGFβ1-treated ARPE-19 cells was significantly increased by 164.81% at 24 h and 415.22% at 48 h after co-treatment with FH535 at a concentration of 0.5 µmol/L (n > 20, *p* < 0.00001, Fig. 5D and E). Collectively, these findings indicated that the activation of the Wnt/β-catenin signaling pathway played a pivotal role in mediating the TGFβ1-induced EMT phenotype of ARPE-19 cells, encompassing the alterations in cell morphology as well as the enhanced cell migration and invasion capabilities.

### Inhibition of Wnt/β-catenin signaling mitigated the increase of Wnt5a expression and ECM deposition in TGFβ1-treated ARPE-19 cells, while the sole activation of Wnt5a stimulated β-catenin and ECM in ARPE-19 cells

**Fig. 6:**
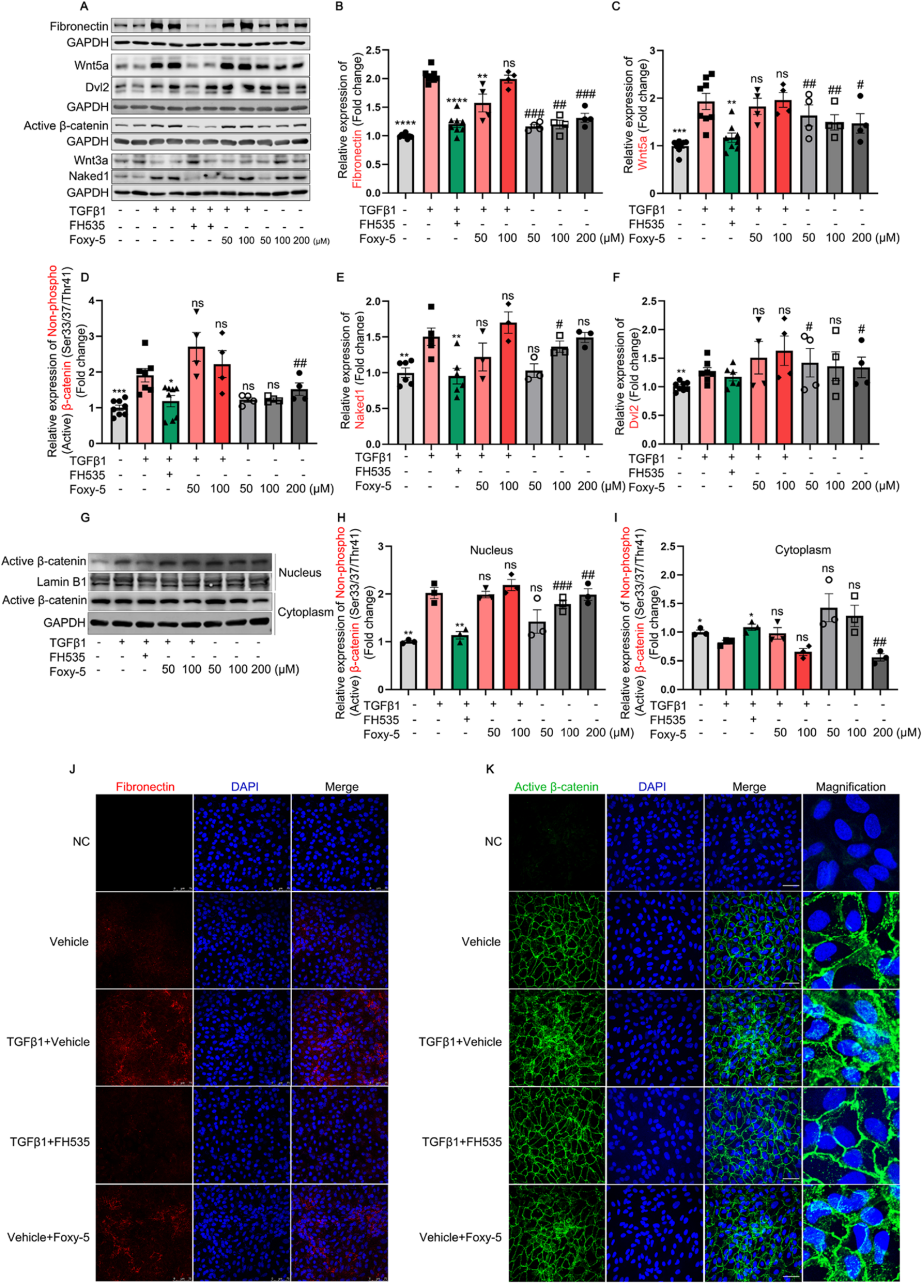
The effects of FH535 (a canonical Wnt signaling inhibitor) and Foxy-5 (a Wnt5a agonist) on Wnt signaling pathway and EMT in ARPE-19 cells. **A** Western blot analysis of fibronectin, Wnt5a, active β-catenin, Naked1, Dvl2, and Wnt3a in ARPE-19 cells treated with TGFβ1 (10 ng/mL) alone or in combination with FH535 (0.5 μmol/L) or Foxy-5 (50 and 100 μmol/L), as well as in untreated ARPE-19 cells with or without Foxy-5 (50, 100, or 200 μmol/L). **B**–**F** Quantitative analysis of Western blot analysis of fibronectin, Wnt5a, active β-catenin, Naked1, and Dvl2 was performed in indicated groups with GAPDH served as loading control. **G**–**I** Expression and quantitative analysis of active β-catenin protein in the nuclear and cytoplasmic extracts of ARPE-19 cells under different conditions; Lamin B1 and GAPDH were used as nuclear- or cytoplasmic-specific protein loading controls, respectively. **J**–**K** The representative images of immunofluorescence for fibrotic marker fibronectin (red) or active β-catenin (green), with DAPI (blue) labeling all nuclei, were obtained from Vehicle (0.005% DMSO), TGFβ1 + Vehicle (0.005% DMSO), TGFβ1 + FH535 (0.5 μmol/L), Vehicle + Foxy-5 (200 μmol/L) groups. *Scale bars* = 75 μm in (**J**) and *Scale bars* = 50 μm in (K). Dvl, dishevelled; NC, negative control; TGFβ1, transforming growth factor beta 1. All results performed above are presented as mean ± SE from three independent experiments. *p < 0.05, **p < 0.01, ***p < 0.001, ****p < 0.0001 compared with TGFβ1-treated ARPE-19 cells; ^#^*p* < 0.05, ^##^*p* < 0.01, ^###^*p* < 0.001 compared with untreated ARPE-19 cells

Subsequently, we sought to elucidate the underlying mechanistic basis for FH535's anti-EMT properties, as well as investigate the potential regulatory interaction between Wnt5a and β-catenin. The results demonstrated that FH535 treatment markedly inhibited the upregulation of fibronectin, Wnt5a, Naked1, and the active β-catenin at the protein level in TGFβ1-treated ARPE-19 cells, while showed no effect on Wnt3a (Fig. 6A-F). The representative immunofluorescence images of fibronectin and active β-catenin are shown in Fig. 6J and K, which confirmed the findings obtained through Western blot analysis.

We next investigated the effect of Foxy-5, a Wnt5a agonist [36], on the EMT and Wnt/β-catenin signaling pathway of ARPE-19 cells. The safe dosage range of this small molecule was determined to be 0–300 μmol/L, as evidenced by the CCK-8 data presented in Additional file 1: Fig. S2C. The results indicated that treatment of ARPE-19 cells with Foxy-5 alone significantly enhanced the protein expression levels of fibronectin, Wnt5a, Dvl2, Naked1 and active β-catenin as determined by Western blot (Fig. 6A–F) and immunofluorescence (Fig. 6J–K). However, the co-treatment of ARPE-19 cells with TGFβ1 and Foxy-5 did not exhibit a synergistic effect on the expression of the aforementioned proteins compared to the treatment with TGFβ1 alone (Fig. 6A–F). To further validate the precise activation status of β-catenin signaling, a nuclear-cytoplasmic fractionation assay was employed to evaluate the translocation of active β-catenin into the nucleus. The data revealed that treatment with TGFβ1 resulted in an upregulation of nuclear expression and a downregulation of cytoplasmic expression of active β-catenin in ARPE-19 cells, compared to the control group. These changes were reversed by co-treatment with FH535. Furthermore, Foxy-5 alone significantly enhanced the nuclear translocation activity of active β-catenin in ARPE-19 cells (Fig. 6G–I). The research findings suggested that the activation of Wnt5a and β-catenin in TGFβ1-treated ARPE-19 cells established a reciprocal interaction loop, known as "Wnt5a/β-catenin," which significantly enhanced the EMT process.

### Inhibition of Wnt5a by Box5 or shRNA counteracts the TGFβ1-induced activation of β-catenin and EMT in ARPE-19 cells

**Fig. 7:**
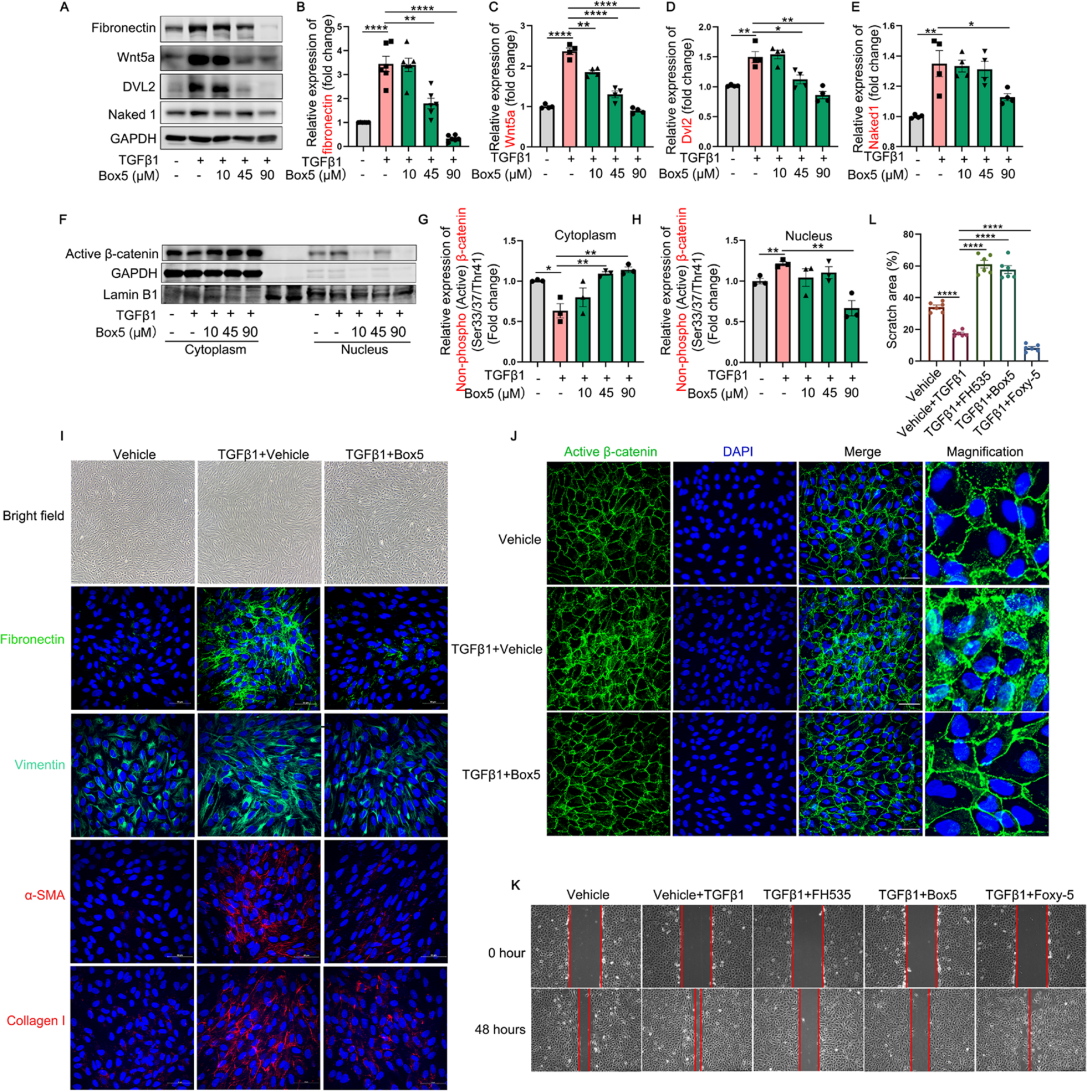
The impact of Box5 (a Wnt5a antagonist) on the expression profiles of EMT- and Wnt signaling-related molecules, as well as its influence on the migratory capacity in TGFβ1-treated ARPE-19 cells. **A**–**E** The protein levels of fibronectin, Wnt5a, Dvl2, and Naked1 in TGFβ1 (10 ng/mL)-treated ARPE-19 cells with or without different concentrations of Box5 (10, 45 and 90 μmol/L) for 48 h. **F**–**H** The expression and quantitative analysis of active β-catenin protein in the nuclear and cytoplasmic extracts of ARPE-19 cells among indicated groups; Lamin B1 and GAPDH were employed as nuclear- or cytoplasmic-specific protein loading controls, respectively. **I**–**J** Bright field images and immunofluorescence analysis of fibronectin, vimentin, α-SMA, collagen I and active β-catenin proteins in ARPE-19 cells among Vehicle (0.9% DMSO), TGFβ1 + Vehicle (0.9% DMSO), and TGFβ1 + Box5 (90 μmol/L) groups. *Scale bars*, 50 μm. **K** and **L** Cell scratch assay was performed to examine the migration and invasion of ARPE-19 cells among different groups. *Scale bars* = 250 μm. α-SMA, alpha-smooth muscle actin; Dvl, dishevelled; TGFβ1, transforming growth factor beta 1. All results performed above are presented as mean ± SE from three independent experiments. *p < 0.05; **p < 0.01, ***p < 0.001, ****p < 0.0001 compared with TGFβ1-treated ARPE-19 cells

To further elucidate the involvement and significance of Wnt5a in activating β-catenin and EMT, a Wnt5a-specific antagonist (Box5) was implemented for subsequent studies by inhibiting Wnt5a signaling, and this small molecule compound exhibits no direct impact on the canonical Wnt/β-catenin signaling [37]. The cell viability of ARPE-19 cells treated with different concentrations of Box5 was examined using the CCK-8 assay, revealing a safe dosage range spanning from 0 to 100 μmol/L (Additional file 1: Fig. S2B). The treatment with Box5 at concentrations of 10, 45, 90 μmol/L suppressed the TGFβ1-mediated up-regulation of fibronectin, Wnt5a, Dvl2, Naked1 in a dose-dependent manner (Fig. 7A–E). For morphological analysis, the bright field images demonstrated the stretched and elongated appearance of ARPE-19 cells following TGFβ1 treatment was significantly reversed upon co-incubation with Box5 (Fig. 7I). Immunostaining of EMT-related markers including fibronectin, vimentin, α-SMA and collagen I further validated that Box5 treatment was able to reduce the fibrosis-related markers caused by TGFβ1 in ARPE-19 cells (Fig. 7I).

Importantly, the results from nuclear-cytoplasmic separation experiments depicted in Fig. 7F–H demonstrated that treatment with TGFβ1 led to a reduction in cytoplasmic expression levels of active β-catenin and an increase in its nuclear expression levels in ARPE-19 cells compared to the control group. Treatment with Box5 effectively attenuated the nuclear translocation activity of active β-catenin in TGFβ1-treated ARPE-19 cells. Moreover, immunofluorescence staining of active β-catenin (Fig. 7J) revealed a significant enhancement in its colocalization with the cell nucleus upon TGFβ1 treatment, which was reversed by co-treatment with Box5.

Subsequently, the scratch assay was performed to evaluate the impact of Wnt5a on cell invasion and migration in TGFβ1-treated ARPE-19 cells. Co-administration of FH535 or Box5 with TGFβ1 exhibited a significantly reduced migratory capacity compared to TGFβ1 alone, while co-incubation of Foxy-5 and TGFβ1 further augmented wound closure and cell migration in ARPE-19 cells (Fig. 7K and L).

**Fig. 8:**
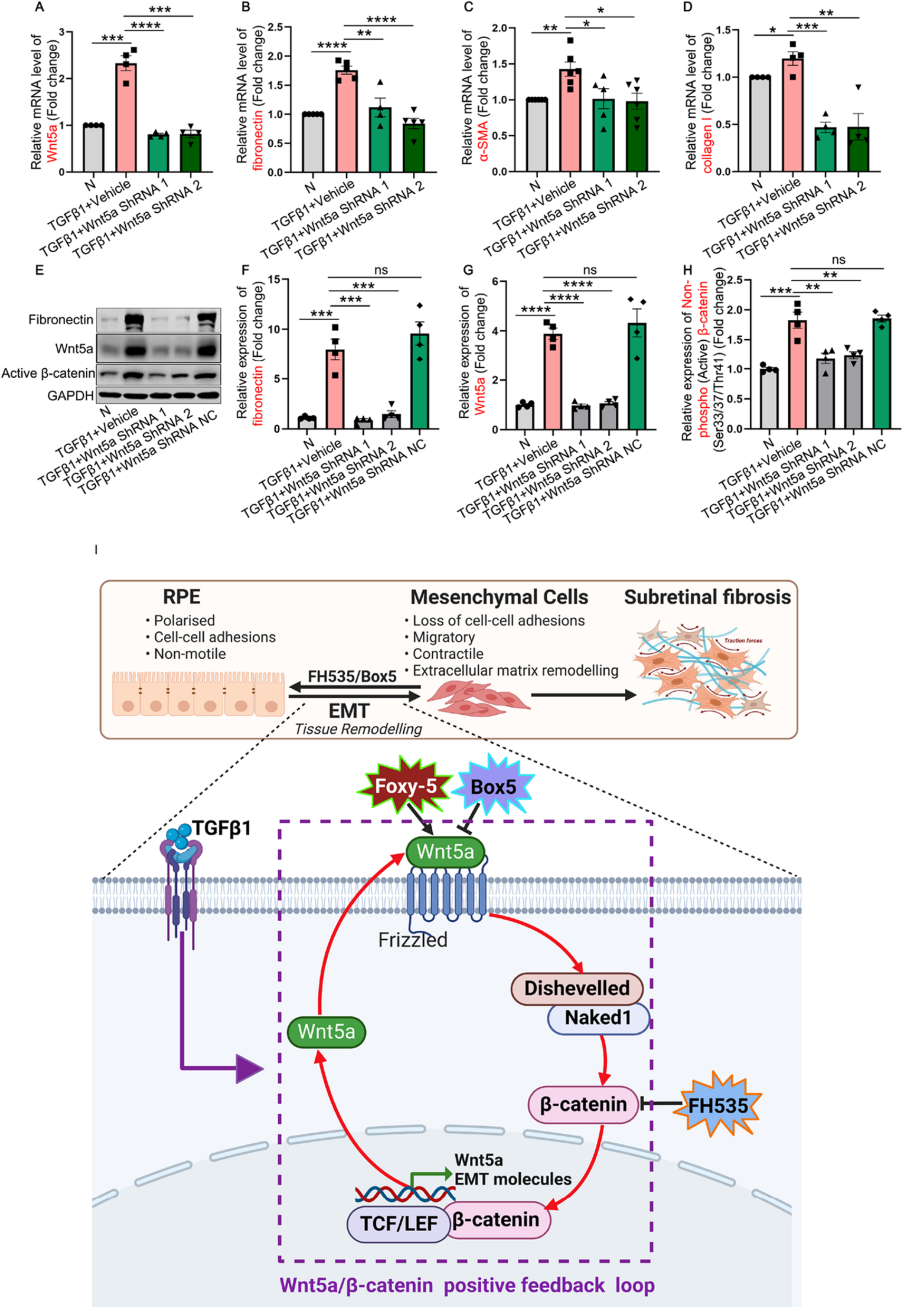
Validation of the role of Wnt5a in regulation of EMT and β-catenin signaling. **A**–**H** ARPE-19 cells expressing control shRNA, Wnt5a shRNA-1 or -2 were treated with TGFβ1 (10 ng/mL) for 48 h. **A**–**D** qRT-PCR and/or **E**–**H** Western blot was employed to examine the mRNA and protein expressions of Wnt5a, fibronectin, α-SMA, collagen I and active β-catenin. **I** Schematic diagram illustrating the positive feedback loop of Wnt5a/β-catenin involved in the process of EMT of RPE cells and subretinal fibrosis secondary to nAMD. The TGFβ1-induced upregulation of the non-canonical Wnt ligand Wnt5a and activation of canonical β-catenin signaling, forming a reciprocal activation, potentially amplifying disease signals and contributing to EMT and subretinal fibrosis. Line arrows indicate activation, whereas connector lines imply inhibition. **I** was created with https://www.biorender.com/. Dvl, Dishevelled; EMT, epithelial–mesenchymal transition; LEF, lymphoid enhancer-binding factor; ROR1, receptor tyrosine kinase-like orphan receptor 1; TCF, T-cell factor; TGFβ1, transforming growth factor beta 1

To further verify the crucial role of Wnt5a in EMT, shRNA was employed to suppress Wnt5a expression to validate the aforementioned results. In TGFβ1-treated ARPE-19 cells, two highly effective Wnt5a shRNAs were capable of reducing Wnt5a expression to the level of control cells (Fig. 8A, E and G). Consistently, ARPE-19 cells transfected with Wnt5a shRNA exhibited a significant downregulation in the mRNA and protein expression of mesenchymal and fibrotic-associated markers, such as fibronectin, α-SMA, and collagen I, as well as the active β-catenin upon TGFβ1 stimulation when compared to TGFβ1-induced ARPE-19 cells (Fig. 8B–F and H).

## Discussion

Subretinal fibrosis, as the most prevalent natural sequelae of nAMD, causally impacts the photoreceptors, RPE, and choriocapillaris, thereby leading to irreversible central vision loss [38]. Despite extensive research on the pathogenesis of nAMD, the molecular mechanisms underlying subretinal fibrosis remain insufficiently characterized. Although the involvement of canonical Wnt/β-catenin activation in subretinal fibrosis has been reported [12, 39], the role of the non-canonical ligand Wnt5a in EMT and subretinal fibrosis remains obscure.

In the present investigation, we observed a significant upregulation of Wnt5a expression in both in vivo and in vitro models simulating subretinal fibrosis or EMT (Fig. 3A and Fig. 4B). Above molecular and phenotypic changes were evidently inhibited by inactivating β-catenin using FH535 (Figs. 2, 3, 5 and 6, and Additional file 1: Fig. S3), thereby corroborating the indispensable role of the canonical Wnt/β-catenin signaling in facilitating EMT [12], as well as its transcriptional effect on the non-canonical ligand Wnt5a. The action of the small molecule FH535 is achieved by interfering with the nuclear translocation of β-catenin in the canonical Wnt signaling pathway, thereby impeding its entry into the cell nucleus and subsequent binding with transcription factors, ultimately resulting in the reduction of downstream target gene expression [40], such as Wnt5a [41]. Furthermore, the inhibition of Wnt5a through Box5 or shRNA could notably reverse the activation of β-catenin, EMT, and subretinal fibrosis both in vivo and in vitro (Figs. 3, 7 and 8). The causative role of Wnt5a in promoting EMT and its positive regulation on β-catenin could be further reinforced in a Wnt5a agonist assay, demonstrating that Wnt5a alone is sufficient to activate β-catenin and initiate EMT (Fig. 6). Based on the aforementioned data, activation of Wnt5a and β-catenin establishes a mutually reinforcing interaction loop involving "Wnt5a/β-catenin" significantly promoting the EMT process and subsequent subretinal fibrosis (Fig. 8I). However, the activation sequence of these two pathogenic factors in the development of EMT or fibrosis remains unclear.

The expression of Wnt5a in myofibroblasts was reported to be induced by the profibrotic factor TGFβ, playing a crucial role in the regulation of fibrotic matrix proteins induced by TGFβ in the context of liver fibrosis. This effect was reversed after silencing Wnt5a [42]. It was also reported that TGFβ-mediated regulation of Wnt5a was confirmed in primary cells, with Smad binding sites identified within the Wnt5a promoter [43, 44]. Thus, it is possible to propose that TGFβ1 mediates the upregulation of Wnt5a transcription, promoting the activation of β-catenin, which ultimately contributes to subsequent EMT and subretinal fibrosis. The precise mechanism underlying TGFβ1-induced upregulation of Wnt5a requires further exploration in the context of subretinal fibrosis secondary to nAMD.

The intricate Wnt signaling system plays a pivotal role in regulating the processes implicated in the pathogenesis of EMT as well as various fibrotic diseases. Although the canonical and non-canonical Wnt signaling pathway are two independent cellular signaling pathways, they can interact and cross-regulate each other in cell development, tissue repair, and disease processes [45]. Wnt5a is recognized to stimulate non-canonical signaling independent of β-catenin [46]. Nonetheless, this non-canonical Wnt ligand can trigger the activation of the canonical Wnt signaling pathway [47, 48]. For example, Wnt5a overexpression in certain cases resulted in an enhancement of TCF/ lymphoid enhancer-binding factor (LEF) transcriptional activity and direct activation or cooperation with the Wnt/β-catenin pathway [49]. Additionally, one study demonstrated that myometrial cells, acting as niche components, regulate the self-renewal activity of endometrial mesenchymal stem-like cells (eMSCs) via Wnt5a-dependent activation of the Wnt/β-catenin signaling pathway [49]. Meanwhile, in specific circumstances, the activation of the canonical Wnt/β-catenin signaling pathway can induce the binding of the transcription factor TCF/LEF to the promoter region of Wnt5a gene, thereby enhancing its transcription [50]. One of the limitations of this study is that we have not yet determined the direct transcriptional impact of β-catenin on Wnt5a, which may necessitate the use of a ChIP-qPCR technique in our future work.

After proposing and validating the concept of the "Wnt5a/β-catenin" interaction loop, we also discovered that FH535, Box5, and Wnt5a shRNA displayed comparable inhibitory effects on subretinal fibrosis and EMT (Figs. 3 and 5–8). In other words, either the blockage of Wnt5a or β-catenin could disrupt this interaction loop (Fig. 8). Notably, although no apparent changes were observed in the expression of Wnt3a and Wnt1 both in vivo and in vitro (Figs. 3A and 4A), it is important to acknowledge that the canonical Wnt/β-catenin signaling pathway is not solely regulated by these ligands alone. Therefore, we cannot disregard the potential involvement of additional Wnt ligands in β-catenin activation within the scope of this study. One research validated our findings by demonstrating elevated level of Wnt5a and unaltered expression of wnt3a in laser-treated RPE compared to control RPE, as determined through RT-qPCR analysis. However, this study also identified significant increases in other Wnt ligands, such as Wnt1, Wnt2b, Wnt3, Wnt7a, Wnt7b, and Wnt10b, which might also contribute to the activation of β-catenin [12]. Furthermore, we cannot exclude the potential involvement of the Wnt5a-mediated non-canonical Wnt signaling activation in the pathogenesis of EMT [51, 52] and subretinal fibrosis, which also merits further study.

The underlying mechanisms of subretinal fibrosis secondary to nAMD are complicated and remain incompletely elucidated. Extensive literature corroborated the involvement of additional factors, such as pericyte-myofibroblast transition (PMT) [53], EndMT [54], activated microglia [55], macrophages [56] and Müller glia [57] in the laser-induced CNV mouse model, which contribute to the accumulation of differentiated myofibroblasts and deposition of ECM, ultimately leading to fibrosis formation in nAMD. Therefore, the contribution of Wnt5a or Wnt/β-catenin in the MT of other cell types during subretinal fibrosis remains to be elucidated. Notably, the critical role of EMT undergone by RPE cells during subretinal fibrosis has been extensively recognized and corroborated [58, 59]. The intricate molecular networks governing EMT exhibit interconnectedness and reciprocal interactions. This process involves the activation of not only the Wnt signaling but also the canonical TGF-β-Smad signaling, as well as the non-canonical TGF-β signaling pathways, including phosphatidylinositol-3-kinase/Akt (PI3K/Akt), Rho/Rho kinase (ROCK), mitogen-activated protein kinases (MAPKs), Jagged/Notch, Hedgehog (Hh), and other signaling pathways [60]. It is proposed that patients with nAMD who exhibit an inadequate response to anti-VEGF therapy could potentially benefit from a combined therapeutic approach that targets both VEGF and the EMT process of RPE cells within the fibrovascular scar, thereby achieving a more favorable prognosis.

Subretinal fibrosis often occurs concurrently with neovascularization in nAMD, as exemplified by the co-localization of collagen I and IB4 in the CNV lesions visualized by confocal microscope in Fig. 2C and D. The early intervention of pathological neovascularization potentially mitigates the infiltration of immune cells and inflammatory response, thereby facilitating the prevention of subsequent subretinal fibrosis [61]. Despite its seemingly dual-functionality in physiological angiogenesis, Wnt5a signaling is essential for pathological angiogenesis [62]. Experimental studies on various vascular eye diseases, including nAMD, diabetic retinopathy (DR), retinopathy of prematurity (ROP), and corneal neovascularization, indicate that an aberrantly heightened Wnt signaling pathway is among the potential causative factors for pathological ocular neovascularization [17]. The activation of the Wnt pathway has been reported in laser-induced CNV models, indicating its pathogenic role in CNV. Inhibition of Wnt signaling through the use of an anti-LRP6 antibody demonstrates therapeutic potential for treating CNV [63]. Therefore, it is difficult to assert that the attenuation of subretinal fibrosis through inhibition of Wnt5a/β-catenin signaling pathway partially stemmed from its inhibitory impact on CNV, which merits further investigation. In order to mitigate the confounding effect of CNV and subretinal fibrosis, we administered the inhibitor injection at a later time point (Day 14) after laser induction in mice. This decision was based on the report that neovascularization in this mouse model reaches its peak stage at day 7 and progresses gradually [64]. Remarkably, our findings demonstrate that inhibition of Wnt5a/β-catenin pathway remained highly effective in combating fibrosis and EMT during the late stage (Day 21) of this mouse model (Fig. 3).

These findings and discussion should be taken into account when developing specific drugs for the treatment of subretinal fibrosis, as only targeting the mesenchymal transition and activation of multiple retinal cells is insufficient. Instead, a combination approach with inhibition of pathological neovascularization should be employed to effectively inhibit both the neovascularization and subretinal fibrosis. In developed countries, a significant proportion of vision impairment arises from abnormalities in the retinal or choroidal vasculature. These conditions, encompassing ailments such as nAMD [2], DR [65], ROP [66], and neovascular glaucoma [67], share the hallmark features of macular edema, retinal and vitreous hemorrhage, or the formation of fibrovascular scars. A shared underlying mechanism in these diverse conditions lies within the retina's response to damage, eventually setting off an ongoing cycle of chronic wound healing that may culminate in fibrotic tissue formation and, in the worst cases, irreversible vision impairment [68]. Therefore, the mechanistic insights gleaned from the nAMD study and the combined inhibition of subretinal fibrosis and neovascularization mentioned above has important clinical significance in developing a treatment for the prevention of the subretinal fibrosis and neovascularization in other ocular diseases.

In summary, our research findings unequivocally demonstrated the pivotal role of the positive interaction loop of Wnt5a/β-catenin in inducing EMT of RPE cells, facilitating cell migration, and contributing to subretinal fibrosis in nAMD, as illustrated in the schematic diagram presented in Fig. 8I. Furthermore, exploring the involvement of Wnt5a in the pathogenesis of AMD-related conditions beyond EMT, such as EndMT, PMT, and glial cell activation, holds promising potential for further exploration. Moreover, considering the pivotal role of the Wnt signaling pathway in pathological neovascularization across diverse diseases, it is plausible to speculate that Wnt5a/β-catenin might serve as a key role in driving angiogenesis of RNV and CNV secondary to nAMD. Our findings shed light on one of the intricate molecular pathways underlying subretinal fibrosis and offer novel insights into potential therapeutic strategies for the management of the fibrovascular diseases by targeting Wnt5a/β-catenin-mediated EMT. In future, when developing novel therapies for treating nAMD, these findings should be taken into careful consideration, aiming to employ minimal drug combinations to address the pathological neovascularization, subretinal fibrosis, inflammation, and the various cellular processes involved in EMT or activation within the retina. This approach seeks to optimize the therapeutic strategy for the management of nAMD and other fibrovascular diseases.

### Supplementary Information

**Additional file 1:** **Figure S1.** IB4 (green) and DAPI (blue) staining of 4-week retinal sections showed that RPE cells appeared to surround the pathological choroidal neovascularization resulting from Bruch membrane’s rupture due to subretinal injection. CCE: choroidal capillaries; IB4, isolectin B4; RPE: retinal pigment epithelium. *Scale bars* = 200 μm. **Figure S2.** The effect of FH535, Box5 and Foxy-5 on the cell viability of ARPE-19 cells. The cell viability of ARPE-19 cells examined with CCK-8 assay after treatment with the indicated doses of (A) FH535, (B) Box5 or (C) Foxy-5 for 48 h. The Vehicle Control groups for **A** FH535 and **B** Box5 contained DMSO concentrations of 0.2% and 2%, respectively. (Data are expressed as mean ± SEM. *p < 0.05, **p < 0.01, ***p < 0.001, ****p < 0.0001 compared with untreated ARPE-19 cells). **Figure S3.** The inhibitory effect of FH535 on EMT in TGFβ1-induced ARPE-19 cells. **A**–**F** mRNA and **G**–**I** protein levels of EMT-related markers, including **A**, **G** fibronectin, **B** collagen I, **C**, **H** α-SMA, **D** Snail 1, **E** TAGLN, **F** MMP2, as well as epithelial marker. **I** ZO-1 measured by qRT-PCR and Western blot, respectively, in TGFβ1-treated ARPE-19 cells for 48 h with or without different concentrations of FH535 treatment. α-SMA, alpha-smooth muscle actin; MMP2, matrix metallopeptidase 2; TAGLN, transgelin; TGFβ1, transforming growth factor beta 1; ZO-1, zonula occludens-1. Data are expressed as mean ± SEM. *p < 0.05, **p < 0.01, ***p < 0.001, ****p < 0.0001 compared with the TGFβ1-treated group. **Figure S4.** Safety assessment of intravitreal administration of Box5 in C57 mice. **A**–**C** Representative results of scotopic ERG at 0.01 or 3.0 log cds/m^2^ and photopic ERG at 3.0 log cds/m^2^ in 4-month-old male C57BL/6J mice 7 days after intravitreal injection of Box5 (90 μmol/L), compared to the Vehicle group injected with PBS. **D** The mean scotopic ERG b-wave amplitudes elicited by 0.01 log cds/m^2^ white-light stimuli. **E** The mean scotopic ERG a-wave amplitudes elicited by 3.0 log cds/m^2^ white-light stimuli. **F** The mean scotopic ERG b-wave amplitudes elicited by 3.0 log cds/m^2^ white-light stimuli. **G** The mean photopic ERG b-wave amplitudes elicited by 3.0 log cds/m^2^ white-light stimuli. ERG, Electroretinography; Data are expressed as mean ± SEM. ns, no significant.

## Data Availability

The original data will be transferred and shared following the instruction by the Editorial Committee.

## Abbreviations

α-SMA: Alpha-smooth muscle actin
AMD: Age-related macular degeneration
AMPK: AMP-activated protein kinase
bFGF: Basic fibroblast growth factor
BSA: Bovine serum albumin
CCK-8: Cell counting kit‐8
CNV: Choroidal neovascularization
DAPI: 4′,6-Diamidino-2-phenylindole
DM: Diabetes mellitus
DME: Diabetic macular edema
DMEM: Dulbecco's Modified Eagle Medium
DMSO: Dimethyl sulfoxide
DR: Diabetic retinopathy
Dvl: Dishevelled
ECs: Endothelial cells
ECM: Extracellular matrix
eMSCs: Endometrial mesenchymal stem-like cells
EMT: Epithelial–mesenchymal transition
EndMT: Endothelial-mesenchymal transition
ERG: Electroretinography
FAK: Focal adhesion kinase
FBS: Fetal bovine serum
FZD: Frizzled receptor
GAPDH: Glyceraldehyde-3-phosphate dehydrogenase
H&E: Hematoxylin and eosin
HRECs: Human retinal endothelial cells
IB4: Isolectin B4
INL: Inner nuclear layer
IPL: Inner plexiform layer
ITGA5: Integrin α5
LEF: Lymphoid enhancer-binding factor
MAPK: Mitogen-activated protein kinase
MMP2: Matrix metalloproteinase 2
nAMD: Neovascular age-related macular degeneration
ONL: Outer nuclear layer
OPL: Outer plexiform layer
PBS: Phosphate-buffered saline buffer
PCP: Planar cell polarity
PFA: Paraformaldehyde
PI3K/Akt: Phosphatidylinositol-3-kinase/Akt
PMT: Pericyte-myofibroblast transition
qRT-PCR: Quantitative real-time polymerase chain reaction
RBCC: RPE-Bruch’s membrane-choriocapillaris complex
RIPA: Radio immunoprecipitation assay lysis buffer
RNV: Retinal neovascularization
ROCK: Rho kinase
ROP: Retinopathy of prematurity
ROR1: Receptor tyrosine kinase-like orphan receptor 1
RPE: Retinal pigment epithelium
RT-qPCR: Real-time quantitative PCR
SFM: Serum-free medium
shRNA: Short hairpin RNA
Snail1: Snail family transcriptional repressor 1
SPF: Specific pathogen-free
TBST: Tris-buffered saline with Tween-20
TCF: T-cell factor
TGFβ: Transforming growth factor beta
TAGLN: Transgelin
VEGF: Vascular endothelial growth factor
Wnt: Wingless/int
ZO-1: Zonula occludens-1
