# Supplementary material for: Wnt5a/β-catenin-mediated epithelial-mesenchymal transition: a key driver of subretinal fibrosis in neovascular age-related macular degeneration
Source: J Neuroinflammation. 2024 Mar 26;21:75. doi: 10.1186/s12974-024-03068-w (PMC10967154; doi:10.1186/s12974-024-03068-w)
Supplement: Supplementary file 1 — Additional file 1: Figure S1. IB4 (green) and DAPI (blue) staining of 4-week retinal sections showed that RPE cells appeared to surround the pathological choroidal neovascularization resulting from Bruch membrane’s rupture due to subretinal injection. CCE: choroidal capillaries; IB4, isolectin B4; RPE: retinal pigment epithelium. Scale bars = 200 μm. Figure S2. The effect of FH535, Box5 and Foxy-5 on the cell viability of ARPE-19 cells. The cell viability of ARPE-19 cells examined with CCK-8 assay after treatment with the indicated doses of (A) FH535, (B) Box5 or (C) Foxy-5 for 48 h. The Vehicle Control groups for A FH535 and B Box5 contained DMSO concentrations of 0.2% and 2%, respectively. (Data are expressed as mean ± SEM. *p < 0.05, **p < 0.01, ***p < 0.001, ****p < 0.0001 compared with untreated ARPE-19 cells). Figure S3. The inhibitory effect of FH535 on EMT in TGFβ1-induced ARPE-19 cells. A–F mRNA and G–I protein levels of EMT-related markers, including A, G fibronectin, B collagen I, C, H α-SMA, D Snail 1, E TAGLN, F MMP2, as well as epithelial marker. I ZO-1 measured by qRT-PCR and Western blot, respectively, in TGFβ1-treated ARPE-19 cells for 48 h with or without different concentrations of FH535 treatment. α-SMA, alpha-smooth muscle actin; MMP2, matrix metallopeptidase 2; TAGLN, transgelin; TGFβ1, transforming growth factor beta 1; ZO-1, zonula occludens-1. Data are expressed as mean ± SEM. *p < 0.05, **p < 0.01, ***p < 0.001, ****p < 0.0001 compared with the TGFβ1-treated group. Figure S4. Safety assessment of intravitreal administration of Box5 in C57 mice. A–C Representative results of scotopic ERG at 0.01 or 3.0 log cds/m2 and photopic ERG at 3.0 log cds/m2 in 4-month-old male C57BL/6J mice 7 days after intravitreal injection of Box5 (90 μmol/L), compared to the Vehicle group injected with PBS. D The mean scotopic ERG b-wave amplitudes elicited by 0.01 log cds/m2 white-light stimuli. E The mean scotopic ERG a-wave amplitudes elicited by 3.0 log cds/m2 [file 12974_2024_3068_MOESM1_ESM.docx]

**Supplementary figures and figure legends**


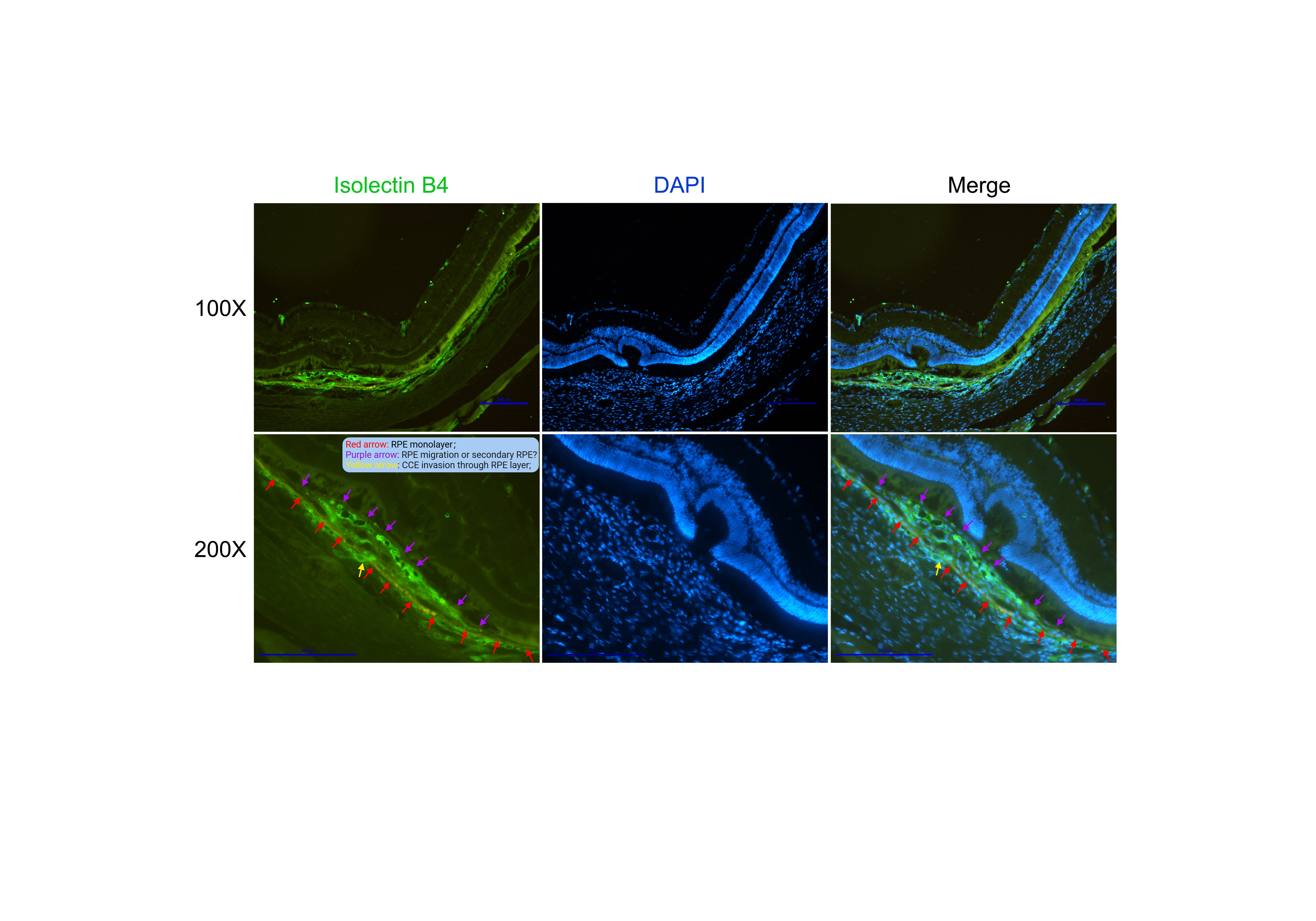
**Fig. S1.** IB4 (green) and DAPI (blue) staining of 4-week retinal sections showed that RPE cells appeared to surround the pathological choroidal neovascularization resulting from Bruch membrane’s rupture due to subretinal injection. CCE: choroidal capillaries; IB4, isolectin B4; RPE: retinal pigment epithelium. *Scale bars* = 200 μm.


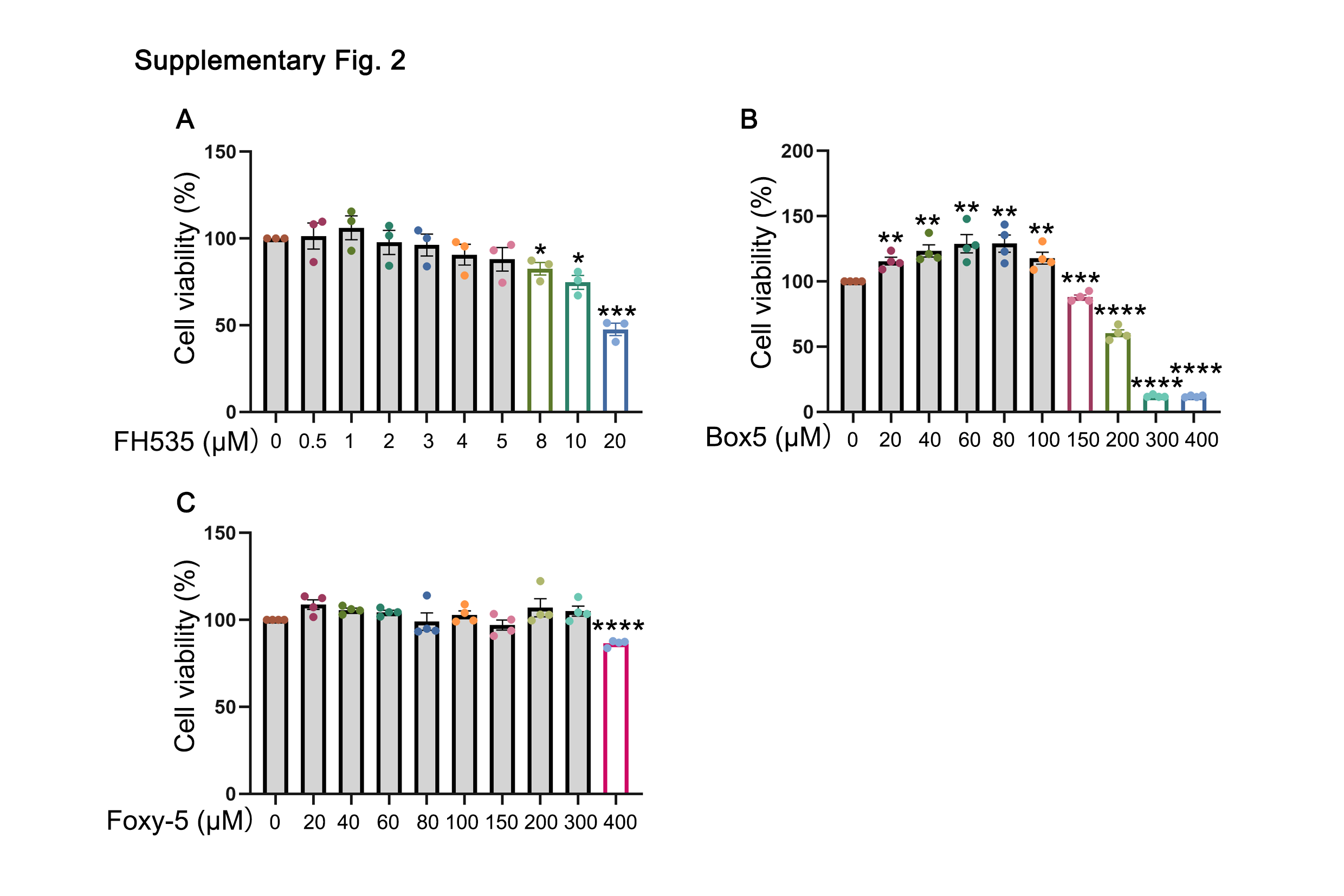


**Fig. S2.** The effect of FH535, Box5 and Foxy-5 on the cell viability of ARPE-19 cells. The cell viability of ARPE-19 cells examined with CCK-8 assay after treatment with the indicated doses of (A) FH535, (B) Box5 or (C) Foxy-5 for 48 hours. The Vehicle Control groups for (A) FH535 and (B) Box5 contained DMSO concentrations of 0.2% and 2%, respectively. (Data are expressed as mean ± SEM. * p < 0.05, ***p* < 0.01, ****p* < 0.001, **** p < 0.0001 compared with untreated ARPE-19 cells).


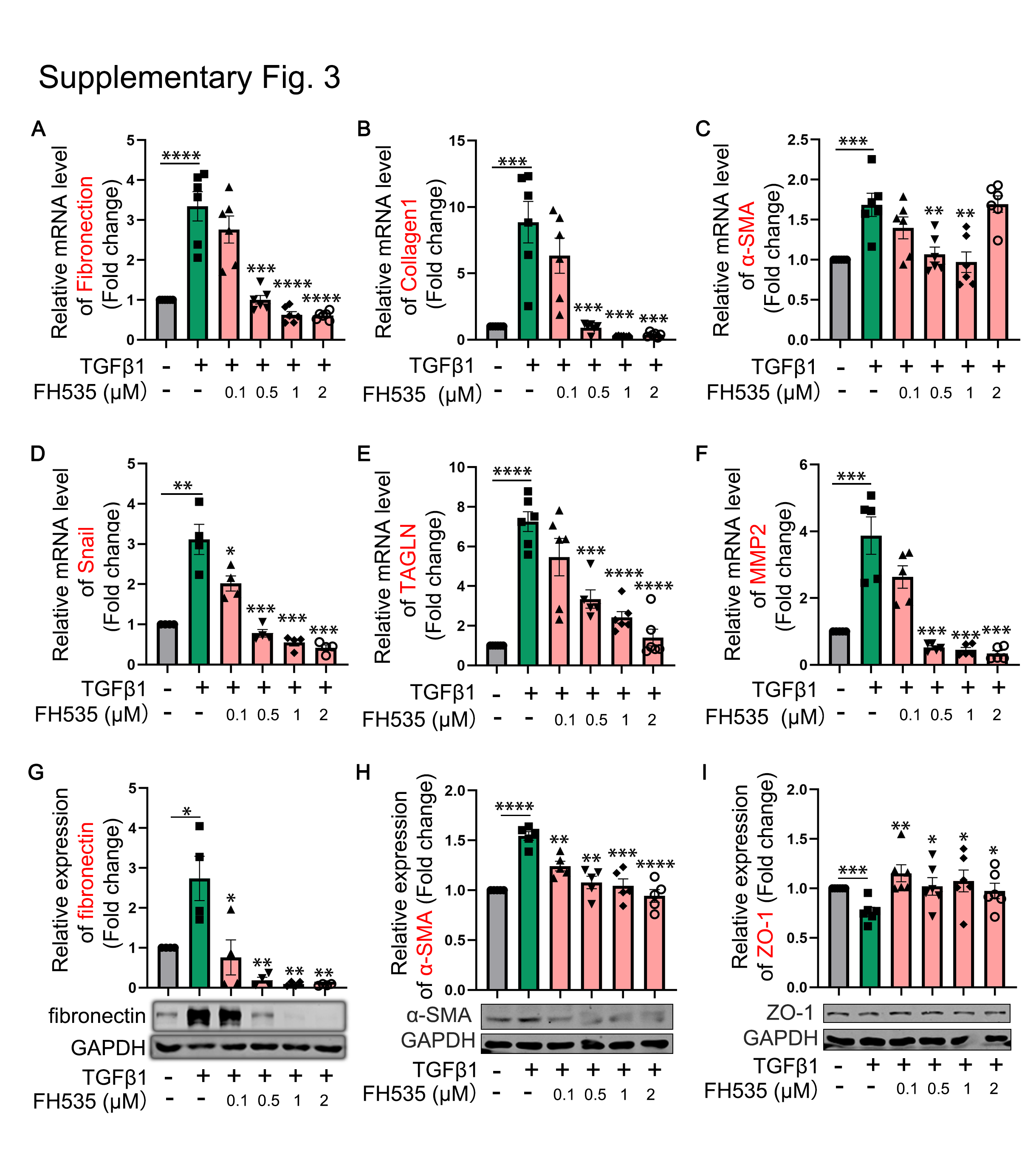


**Fig. S3.** The inhibitory effect of FH535 on EMT in TGFβ1-induced ARPE-19 cells. (A-F) mRNA and (G-I) protein levels of EMT-related markers, including (A, G) fibronectin, (B) collagen I, (C, H) α-SMA, (D) Snail 1, (E) TAGLN, (F) MMP2, as well as epithelial marker (I) ZO-1 measured by qRT-PCR and Western blot, respectively, in TGFβ1-treated ARPE-19 cells for 48 hours with or without different concentrations of FH535 treatment. α-SMA, alpha-smooth muscle actin; MMP2, matrix metallopeptidase 2; TAGLN, transgelin; TGFβ1, transforming growth factor beta 1; ZO-1, zonula occludens-1. Data are expressed as mean ± SEM. * *p* < 0.05, ** *p* < 0.01, *** *p* < 0.001, **** *p* < 0.0001 compared with the TGFβ1-treated group.

**Fig. S**
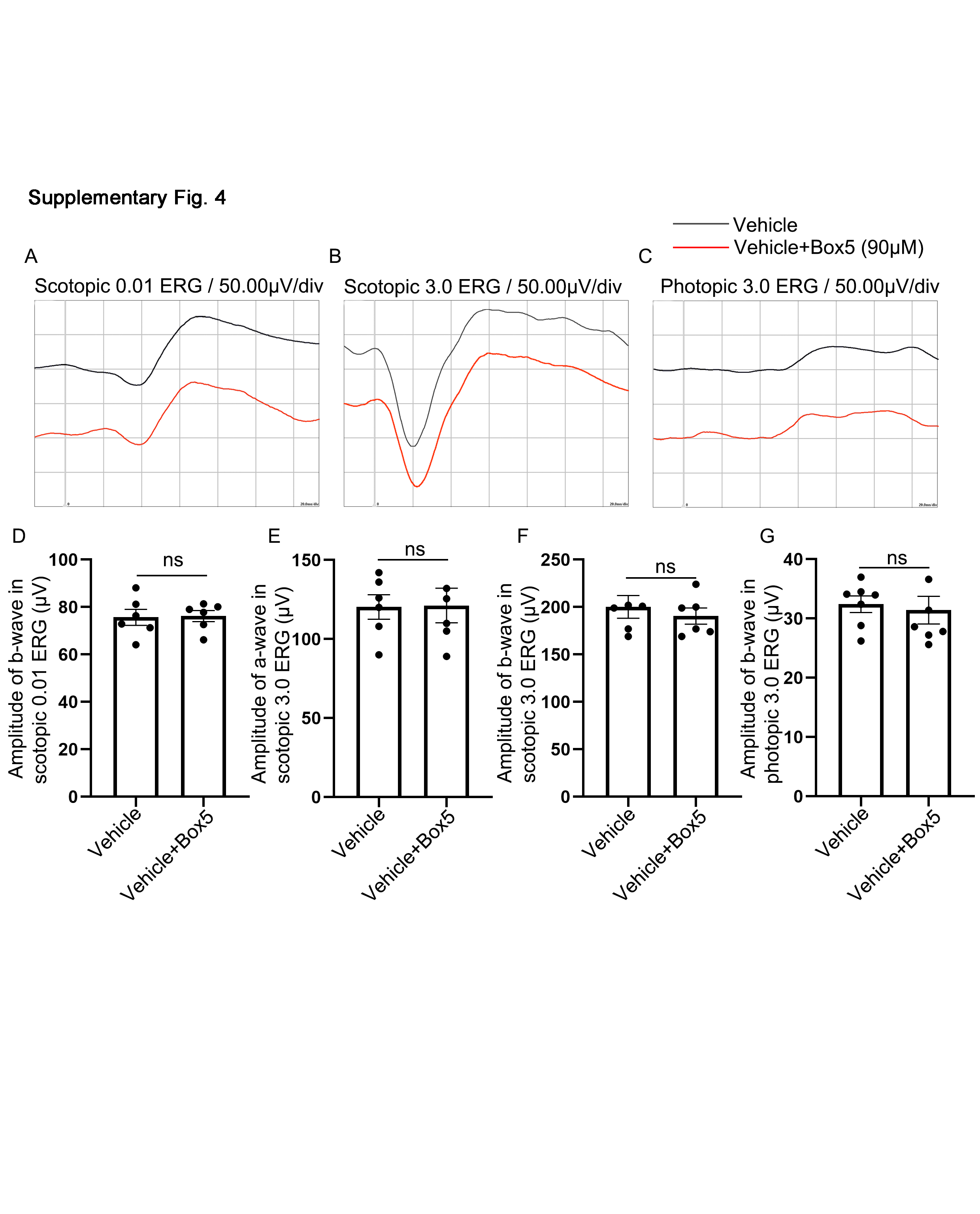
**4.** Safety assessment of intravitreal administration of Box5 in C57 mice. (A-C) Representative results of scotopic ERG at 0.01 or 3.0 log cds/m^2^ and photopic ERG at 3.0 log cds/m^2^ in 4-month-old male C57BL/6J mice 7 days after intravitreal injection of Box5 (90 μmol/L), compared to the Vehicle group injected with PBS. (D) The mean scotopic ERG b-wave amplitudes elicited by 0.01 log cd-s/m^2^ white-light stimuli. (E) The mean scotopic ERG a-wave amplitudes elicited by 3.0 log cd-s/m^2^ white-light stimuli. (F) The mean scotopic ERG b-wave amplitudes elicited by 3.0 log cd-s/m^2^ white-light stimuli. (G) The mean photopic ERG b-wave amplitudes elicited by 3.0 log cd-s/m^2^ white-light stimuli. ERG, Electroretinography; Data are expressed as mean ± SEM. ns, no significant.
